# Expression, Subcellular Localization, and Mechanistic Analysis of Intellectual Disability Syndrome Protein ABBA

**DOI:** 10.1007/s12035-025-05475-3

**Published:** 2025-12-08

**Authors:** Aqsa Jabeen, Pushpa Khanal, Emilia Toissalo, Lauri Lahti, Rimante Minkeviciene, Alexei Kramm, Claudio Rivera, Pirta Hotulainen

**Affiliations:** 1 Minerva Foundation Institute for Medical Research, Helsinki, Finland; 2 University of Helsinki, Helsinki, Finland; 3 Department of Cell Biology, Duke University School of Medicine, Durham, NC USA; 4 Department of Computer Science, Aalto University School of Science, Espoo, Finland; 5 Aix Marseille Univ, INSERM, INMED, Marseille, France

**Keywords:** ABBA/MTSS2, Inhibitory neurons, RAC1, Arp2/3 complex, Dendritic spines, I-BAR domain protein

## Abstract

A missense mutation in the *MTSS2* gene, which encodes the I-BAR domain protein ABBA (Mtss1l/Mtss2), has been linked to an intellectual disability syndrome. To better understand the *MTSS2* mutation-related effect in the brain, we elucidated the cells expressing ABBA and the localization of ABBA in these cells to get insights into which cells and processes might be dysfunctional in mutation-carrying patients. As a novel discovery, we found that ABBA was highly expressed in GABAergic inhibitory neurons, such as parvalbumin-positive interneurons in the hippocampus. At the subcellular level, ABBA localizes to the edges of membrane protrusions in various cells in the brain, suggesting a role in cell migration and spinogenesis. Overexpression of ABBA in pyramidal excitatory and inhibitory neurons increased dendritic spine density. Through live-cell imaging, we demonstrated that ABBA facilitates spine initiation by clustering on the plasma membrane before a new filopodium appears. However, our live cell imaging data also revealed that ABBA localized not only to small focal points, typical for filopodia formation, on the plasma membrane, but also more broadly on the edge of lamellipodial structures. Compared to its close homolog MIM, ABBA appears to be a more general facilitator of protrusion formation, from dendritic filopodia to lamellipodial structures. Altogether, our findings provide insights into ABBA expression, localization, and functional mechanisms, advancing our understanding of its role in neurodevelopmental processes and disorders.

**Supplementary Information:**

The online version contains supplementary material available at 10.1007/s12035-025-05475-3.

## Introduction

ABBA (*Mtss1l/MTSS2*) was initially identified as a regulator of actin and plasma membrane dynamics in radial glial C6 cells [1]. Since its discovery, ABBA has gained broader interest due to two significant findings: first, its mRNA expression increases upon exercise-induced activation of granule neurons in the dentate gyrus of the hippocampus, and it was required for exercise-induced increase in dendritic spine density [2]; second, a missense mutation in *MTSS2*, a human gene encoding ABBA, was found to cause intellectual disability syndrome [3]. This mutation replaces arginine 671 with tryptophan, introducing a bulky aromatic group that destabilizes the protein’s tertiary structure [3].

Patients with the *MTSS2* Arg671Trp mutation exhibit a spectrum of neurodevelopmental symptoms, including global developmental delay, mild intellectual disability, ophthalmological anomalies, microcephaly or relative microcephaly, autism, and distinct facial features [3, 4]. Missing-in-metastasis (*mim*) is a fly ortholog for MIM and ABBA, and *Mim* overexpression with disease-mutation caused similar phenotypes as the loss of *mim* in fly, including shortened lifespan, decreased locomotor activity, bang-sensitivity, and abnormal communication between pre- and postsynaptic cells [3]. Age-related penetrance has been proposed to explain the later onset of severe neurological findings, such as optic atrophy and seizures, in older individuals. This progressive clinical course aligns with findings in model organisms, where the corresponding mutation leads to age-dependent worsening of electrophysiological phenotypes [3].

More detailed knockdown or knock-out experiments at different cell types have demonstrated that ABBA is a multifunctional protein, and the function may change from one cell type to another. Early characterization of ABBA in radial glial C6 cells showed that ABBA knockdown in C6-R cells leads to glial process extension defects [1]. These cells had fewer processes, and the protrusion velocity of lamellipodia of ABBA knockdown cells was slower [1]. In the developing embryonic brain, the loss of ABBA led to a halt in radial glial cell proliferation, disorganized radial fibers, and abnormal migration of neuronal progenitors [5]. Overall, these findings showed that ABBA is necessary for the development of the cerebral cortex by ensuring the accurate progression of mitosis in neuronal progenitor cells. The study also observed a significant decrease in process length in ABBA-knockdown migrating cortical neurons. These changes were rescued by overexpression of wild-type ABBA but not by ABBA-R671W disease-mutation construct [5].

Overexpression of ABBA in hippocampal neurons increased dendritic spine density in vitro and in vivo [2], suggesting a role in the formation of dendritic spines. A single episode of voluntary exercise increased neural activity in a subset of dentate granule cells. The activated cells showed an increase in dendritic spines. shRNA-mediated Mtss2 knockdown in vivo prevented these changes, suggesting that ABBA is required for activity-induced dendritic spine formation in dentate granule cells [2].

Northern blot analysis showed that ABBA is predominantly expressed in the central nervous system (CNS). In situ characterization of mouse embryos further showed that in the brain, ABBA was mainly expressed in radial glia [1]. ABBA was strongly expressed in the floorplate of the spinal cord and hindbrain, and there was weaker expression in parenchyma and in the outer border of the marginal zone where radial glia endplates locate at E12.5 [1]. The floorplate is a transient structure formed by radially oriented glial cells. These results were confirmed with immunohistochemical analysis [1]. In contrast to well-characterized expression in mouse embryos, ABBA expression in young or adult mice is poorly characterized, lacking comprehensive full brain stainings and analyses. In adult mice, the highest levels of ABBA were detected in the molecular layer of the cerebellum, both in the Bergmann glia as well as in the tightly associated Purkinje cell extensions [1]. *Mtss2* mRNA expression could be detected in dentate gyrus granule cells after exercise [2].

ABBA belongs to the I-BAR protein family, characterized by the presence of a BAR (Bin-Amphiphysin- Rvs) domain, which interacts with Phosphatidylinositol 4,5-bisphosphate (PI(4,5)P2)-rich membranes and the actin cytoskeleton to generate membrane curvature. These proteins are essential for neuronal morphogenesis, supporting processes such as neuronal migration, axonal and dendritic extension, and dendritic spine formation [6].

ABBA is an actin-binding protein having a WH2 domain that normally binds actin monomers, not filamentous actin. Actin monomers bind either ATP or ADP. Monomers binding ATP are ‘polymerization-competent’ and ready to be added to the barbed ends of actin filaments. ABBA interacts with ATP-actin monomers with four times higher affinity than ADP-actin monomers [1]. Based on knock-down results, ABBA has a positive effect on protrusion velocity, indicating that it may promote the addition of actin monomers to the barbed ends of actin filaments, next to the plasma membrane in a similar manner to other WH2-domain proteins, such as ciboulot and WASP [1, 7, 8].

ABBA binds Rac1, and deletion of ABBA in C6-R cells markedly inhibited Rac1 activation and cell spreading, suggesting that the interaction between ABBA and activated Rac1 is required for ABBA-promoted cell spreading [1]. Another study further showed that ABBA is activated in response to cell spreading, which markedly promotes cell spreading, and ABBA is required for Rac1 activation and cell spreading [9].

Taken together, in embryonal brain development, ABBA is expressed in radial glial cells, and it contributes to cell division and morphological development of radial glia. Currently, comprehensive characterization of the ABBA expression in the adult brain is lacking. The characterization of the ABBA disease-mutation suggests that the mutated protein is more harmful when expressed than when absent [3]. Therefore, expression of the mutated ABBA should be most harmful in cells that have high ABBA expression. By knowing which cells have high ABBA expression in the adult brain, we should know which cells are mostly affected in the adult brain. The first main objective of this study is to characterize post-natal ABBA expression in the brain and identify cells with high ABBA expression.

As a novel finding, we demonstrated that ABBA is highly expressed in parvalbumin-positive inhibitory neurons in the adult mouse brain. Thus, we expect that from different neuronal subtypes, especially inhibitory neurons, would be affected by ABBA-R671W disease-mutation carriers.

ABBA contributes to cell division and elongation of various types of protrusions. We were mostly interested in the role of ABBA in dendritic spine formation. Based on knock-down studies, the ABBA is required for exercise-induced increase in spine density in dentate gyrus granule cells [2], but the mechanistic details underlying the spine formation are missing. The second main objective of this study is to bring new mechanistic insights into how ABBA contributes to dendritic spine formation. We have earlier characterized mechanistic details for the close homologue of ABBA, MIM, and another spine initiation factor, Gas7, in spine initiation [10, 11], and we hypothesized that ABBA works in spine initiation similarly to MIM. Our mechanistic characterization in hippocampal primary neurons showed that ABBA and MIM share some characteristics, such as PI(4,5)P2 binding and requirement of Arp2/3 complex activity, but overall, ABBA seems to be a more general protrusion inducer compared to MIM, which induced mostly small spine precursor structures. In addition to those structures, ABBA induced broader lamellipodial structures. Interestingly, ABBA enhanced actin depolymerization in the Latrunculin B actin depolymerization assay. We confirmed earlier results of co-localization with inactive and active Rac1 constructs, but in contrast to fibroblasts, where Rac1 activity was required for ABBA-induced protrusion of lamellipodia, Rac1 activity was not required for ABBA-induced increase in dendritic spine density.

## Materials and Methods

### Animals

In the present study, we have used wild-type and transgenic Tg(Thy1-EGFP)MJrs/J mice (Jackson Laboratory) on a C57Bl/6 J background. Tg(Thy1-EGFP)MJrs/J mice express EGFP in sparse subsets of neurons within specific populations. All experimental procedures were carried out according to Finnish laws and ethics under the EU directive 2010/63/EU (licenses: ESAVI/7404/2021, ESAVI/30031/2024, and GMO 3/S/12). Animals were kept in cages as groups of 2–4 mice in a controlled environment (temperature 21 ± 1 °C, humidity 50 ± 10%, light period 06:00 AM to 6:00 PM) and supplied with food and water ad libitum.

For tissue collection, animals were deeply anesthetized with pentobarbital solution (200 mg/kg) containing lidocaine and transcardially perfused using cold PBS. The brains were dissected and kept in 4% PFA for 24 h and then cryoprotected in 20% sucrose solution for at least 48 h before cryo-sectioning. The Thy1-GFP brains were cut sagittally into 50 µm sections with a cryostat (Leica) and stored in cryoprotectant solution (30% ethylene glycol, 20% glycerol in PBS) at −20 °C until further processing.

The wild-type mice left-brain hemisphere was fixed with 4% PFA for 3 days, followed by 70% ethanol for 3 days (daily refreshing solutions), and then paraffinized during a 10-h adult mouse brain program. Then, 5 μm sections were cut for staining. The right brain hemisphere was dissected into the cortex, hippocampus, and cerebellum, which were fast frozen in liquid nitrogen and stored for Western blot at −80 °C.

### Organotypic Slices

RccHan: WIST (Inotiv) rat pups were used for organotypic brain slices. At postnatal days 5–10, rat pups were first anesthetized by using isoflurane and then euthanized by decapitation. Both hippocampi were dissected and sliced transversely into 400 μm cross-sections using a tissue chopper. The slices were placed onto sections of nitrocellulose membrane ~ 5 mm^2^, which were placed on a Millicell cell culture insert with a pore size of 0.4 μm and a diameter of 30 mm on the 6-well plate containing 1.2 ml of culture medium. The culture medium was composed of 95.5 ml Opti-MEM (Gibco), 50 ml of HBSS (Gibco), 50 ml Horse serum (Gibco), and 2 ml of D-glucose (50%), and the pH was set to 7.2. After plating, the hippocampal slices were kept at 37 °C in 5% CO2. At DIV3, anti-mitotic (0.5 mM uridine, 0.5 mM ARA-C, 0.5 mM 5-Fluoro-deoxy-uridine) was added to inhibit the glial growth. At DIV4, the media were replaced with fresh media. Later, the culture media were refreshed twice a week. At DIV10, slices were treated either with 180 ng of BDNF for 24 h or 30 μM Bicuculine for 1 h, diluted in culture media. After treatments, the slices were put back into refreshed culture media and incubated for 4 h, 1 day, and 2 days from the start of the treatment. Slices were then snap-frozen and stored at −80 °C until protein extraction.

### Characterization of ABBA Antibody

**Fig. 1:**
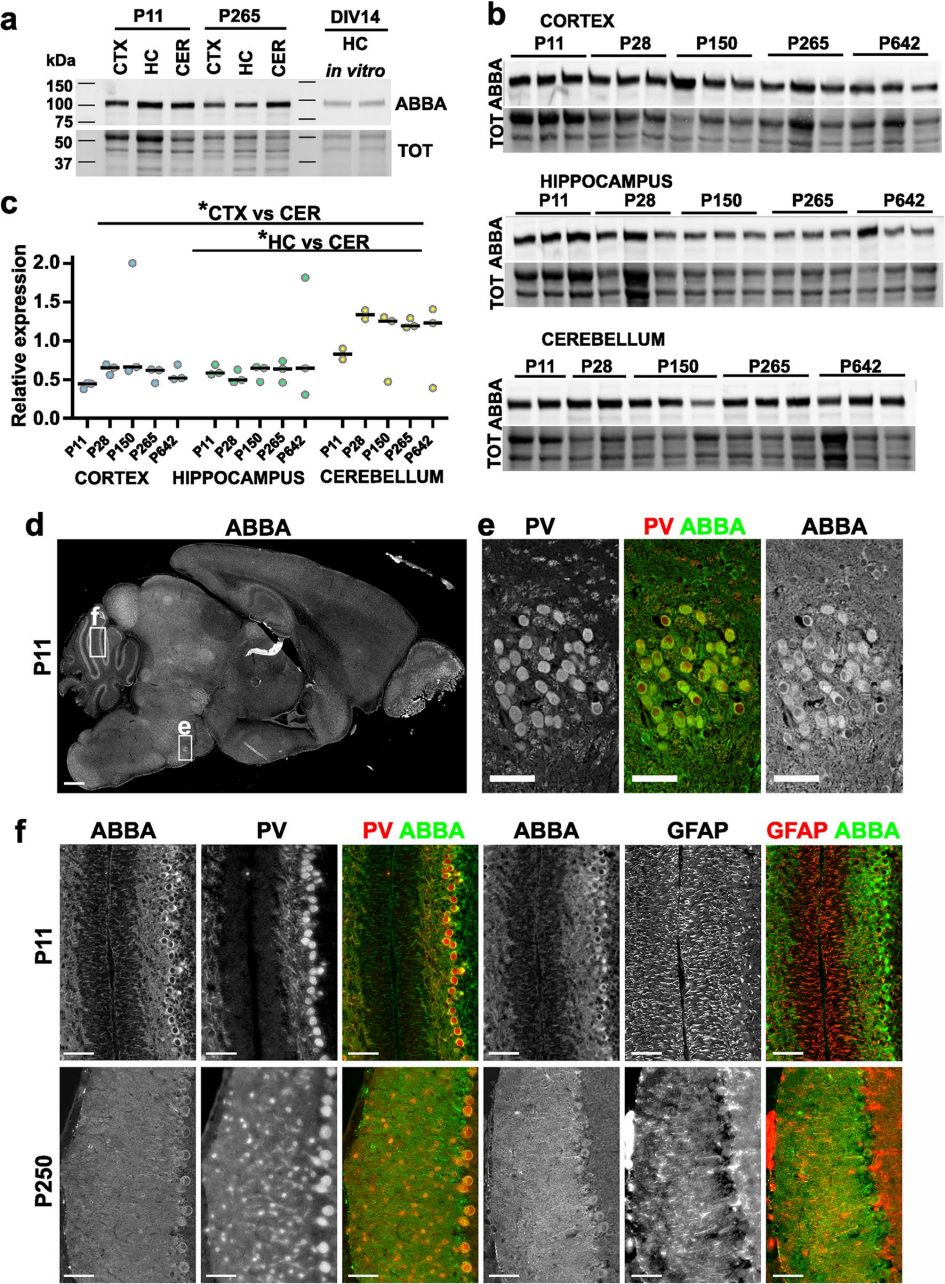
ABBA is expressed in specific cells in the brain. **a** Tissues obtained from cortex (CTX), hippocampus (HC), and cerebellum (CER) of P11 and P265 mice and DIV14 cultured rat hippocampal (HC) neurons were run on SDS-PAGE gel and blotted against ABBA. ABBA protein runs above ~ 100 kDa. Total protein staining is shown below the ABBA Western blot. Uncropped Western blots are shown in Supplementary Fig. 1. **b** Cortex, hippocampus, and cerebellum tissue samples of P11, P28, P150, P265, and P642 mice were run on SDS-PAGE gel and blotted against ABBA. Per age group, 3 animals were used except for P11 and P28 of the cerebellum, only 2 animals were used. **c** The relative expression level of ABBA was quantified against total protein levels. The expression levels are similar in all tested brain regions except in the cerebellum at P250. ABBA expression is higher in the cerebellum at P250 than in the cortex and hippocampus. *p < 0.05, unpaired t-test, Tukey’s multiple comparison test. The grouped graph presents the mean values. **d** The sagittal section of the P11 mouse brain stained with anti-ABBA antibody (ABBA). Scale bar, 500 μm. **e** Zoom in on the superior olivary complex from the D. Sagittal P11 mouse brain slice is stained with anti-Parvalbumin (PV) and anti-ABBA (ABBA) antibodies. Scale bar, 50 μm. **f** Represented images of the cerebellum at P11 and P250 stained with ABBA, PV, and GFAP antibodies. Scale bar, 50 μm

In western blotting and immunostaining, we used an earlier characterized rabbit ABBA antibody. This was originally generated in the Lappalainen laboratory [1] against the central region of mouse ABBA protein that is not conserved in other I-BAR-domain proteins, such as close homologue MIM or IRSp53. This study demonstrated with Western blot analysis that the antibody detected only a single band of expected mobility (around 100 kDa) from brain lysates and in NIH3T3 cells expressing a GFP-fusion protein of ABBA [1]. Furthermore, the antibody did not recognize other I-BAR-domain proteins, IRSp53 and MIM, on a western blot, suggesting that the antibody is specific to ABBA [1]. The antibody was later reproduced in Rivera´s laboratory, and in their recent study, this antibody was used to detect decreased ABBA expression in shRNA ABBA-treated C6 cells, indicating that it is indeed ABBA that the antibody recognizes [5]. Uncropped western blots shown in Fig. 1 are provided as Supplementary Fig. 1.

### Immunostaining/Immunohistochemistry

**Fig. 2:**
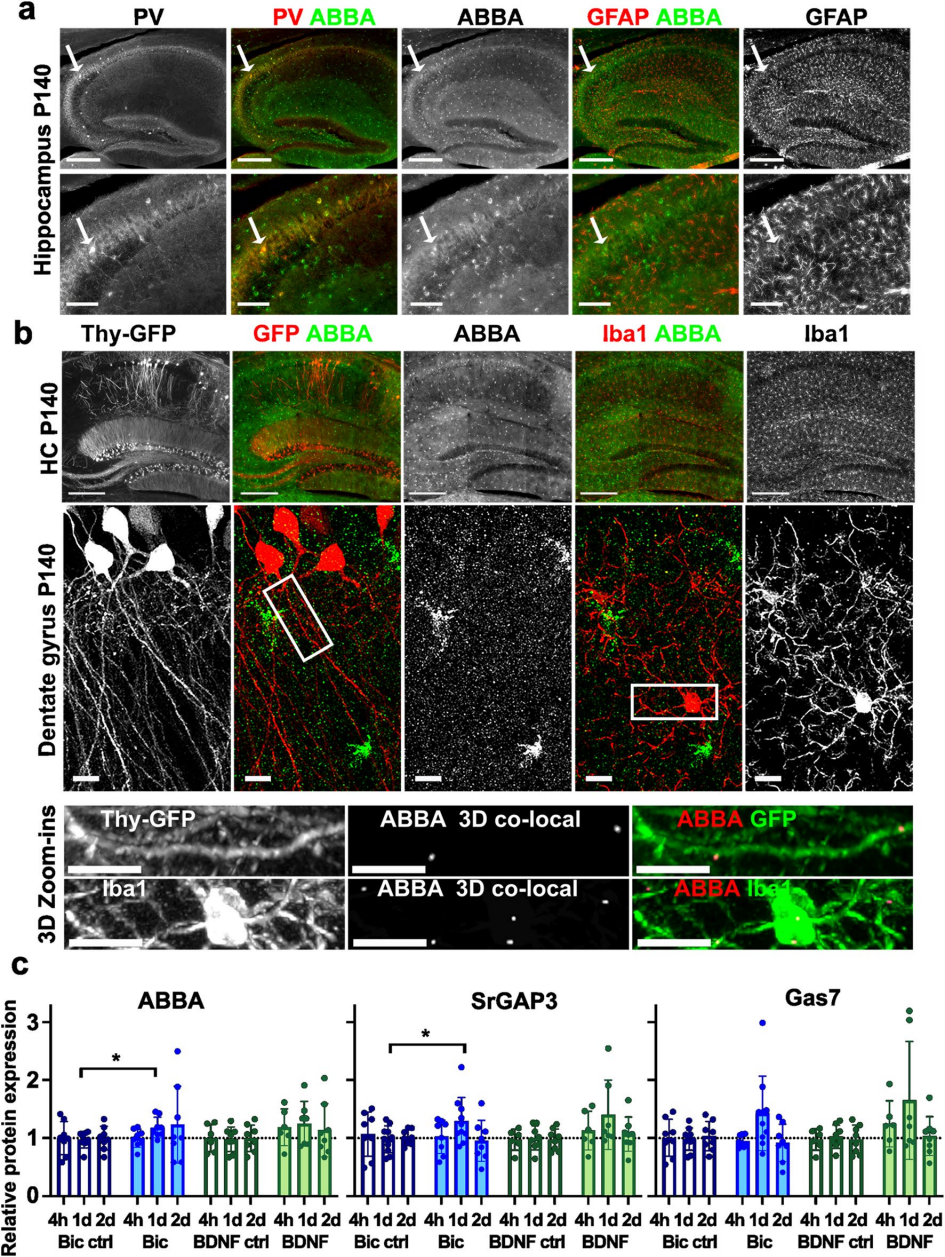
Expression and subcellular localization of ABBA in the hippocampus. **a** The zoomed-in images of the sagittal section of P140 mouse stained with anti-ABBA, anti-parvalbumin (PV, PV-positive inhibitory neurons), and anti-GFAP antibody (astrocytes) staining showing hippocampus (upper panel) and CA2 area in bigger magnification (confocal, lower panel). Scale bars, 300 μm (upper) and 100 μm (lower). Arrows point to the same PV- and ABBA-positive inhibitory neuron. **b** The zoomed-in images of the sagittal section of P140 Thy-GFP transgenic mouse stained with anti-ABBA and anti-Iba1 antibody (microglia) staining showing hippocampus (upper panel) and dentate gyrus area in higher magnification (confocal, middle panel). The lowest panel shows a 3D zoom-in into GFP-positive granule cell dendrite or Iba1-positive microglia, highlighted in the dentate gyrus image with white rectangles. Images in the middle show ABBA-positive dots, which co-localize with GFP or Iba1-staining. Scale bars 300 μm (upper) and 10 μm (middle and lowest). **c** The expression of ABBA, SrGAP3, or Gas7 in DIV10 organotypic brain slices treated with Bicuculline or BNDF for 4 h (4 h), 1 day (1d), or 2 days (2d) was compared to control levels. The relative expression levels of ABBA, n = 4, and SrGAP3, n = 5, increased after 1 day following bicuculine treatment (1 h) compared to controls. *p < 0.05, unpaired t-test

Both paraffin  (5 µm, Fig. 1) and cryosections (50 µm, Fig. 2) were used for stainings. Briefly, paraffinized brain sections went through deparaffinization and rehydration. After antigen retrieval (1 h boiling in 0.01 M citrate buffer pH 6.0), blocking was done by using 2% BSA, 5% milk, 5% sucrose, and 1% Triton X-100 in PBS. Cryosections were first permeabilized with 0.1% Triton X-100 in PBS and then blocked for 1.5 h in a blocking buffer containing 0.3% Triton X-100 and 5% goat serum in PBS.

The sections were then incubated in blocking solution with primary antibodies: rabbit anti-ABBA (1:500 [5], guinea pig polyclonal antiserum against parvalbumin (1:500, Synaptic Systems, 195 004), chicken polyclonal anti-GFAP (1:4000, Abcam, Ab4674), and chicken monoclonal recombinant IgY anti-Iba1 (1:1000, Synaptic Systems, 234 009)COMMENT:These are product numbers and "," look confusing overnight at 4 °C. The next day, sections were incubated in secondary antibodies: anti-rabbit Alexa-488, anti-rabbit Alexa-647 (1:1000, ThermoFisher, A21206, A31573), anti-guinea pig Alexa-568 (1:1000, ThermoFisher, A11075), and anti-chicken Alexa-647 (1:1000, ThermoFisher, A-21449) for 2 h and mounted with glass coverslips using Immu-mount (Thermo Scientific, 9990402). The stained fixed brain slices were scanned using a 3DHISTECH Panoramic 250 FLASH III digital slide scanner using a 20X/0.8 NA objective at the FIMM digital microscopy and molecular pathology unit. For better resolution and 3D images, the brain slices were further imaged through a Zeiss LSM880 inverted confocal microscope using 20X/0.80 NA dry or 63X 1.4 NA oil immersion objectives.

### Western Blotting

The hippocampus, cortex, and cerebellum of wild-type mice were homogenized and lysed in RIPA buffer (50 mM Tris–HCl, pH 7.4, 1% NP-40, 0.25% sodium deoxycholate, 150 mM NaCl, 1 mM EDTA) supplemented with 10% protease inhibitor, 10% phosphatase inhibitor cocktail (Roche), and 1% SDS, using a bead mill homogenizer with 1.4 mm ceramic beads for 15–20 s. Organotypic brain slices were similarly processed with sonication. Protein concentration was measured using the BCA Protein Assay (ThermoFisher Scientific, 23227). For analysis, 20 µg of protein was separated on 10% SDS-PAGE gels and transferred to PVDF membranes. Membranes were blocked for 5 min in Bio-Rad EveryBlot blocking buffer (Bio-Rad, 12010020) and incubated overnight at 4 °C with primary antibodies diluted in blocking buffer: Rabbit anti-ABBA (1:1000 [5], rabbit anti-srGap3 (1:1000, Novus, NBP1-88831), and mouse anti-Gas7 (1:1000, Santa Cruz, SC-365385). Membranes were washed three times with TBS-T and incubated with StarBright secondary antibodies (anti-rabbit, 1:2500, Invitrogen, 1200Y161; anti-mouse, Invitrogen, 12005866) diluted in blocking buffer and 0.02% SDS for 1 h at room temperature. Membranes were again washed three times with TBS-T and imaged using the ChemiDocTM MP Imaging System (Bio-Rad). The protein levels were quantified using Image Lab software (Bio-Rad) and normalized against total protein per lane. Precision Plus Protein Unstained (Bio-Rad, 161–0363) and All Blue Standards (Bio-Rad, 1610373EDU) were used as molecular weight references.

### Hippocampal Cultures and Transfections

The plasmids pmCherry-C1 and pEGFP-C1 were purchased from Clontech Laboratories, Inc. GFP-ABBA and mCherry-ABBA were gifts from Juha Saarikangas. The RFP-LifeAct construct was a gift from Roland Wedlich-Söldner (University of Münster, Münster, Germany) [12]. GFP-LifeAct was a gift from Emmanuel Lemichez (an Institute Pasteur, Université Paris Cité, France). The murine N-WASP-mCherry construct was provided by Maria Vartiainen (Institute of Biotechnology, University of Helsinki). mCherry-actin plasmid was a gift from Martin Bähler (Westfälische Wilhelms-University, Münster, Germany). The Scar W and WA constructs [13] were a gift from Laura Machesky (Beatson Institute for Cancer Research, Glasgow, United Kingdom). mCherry-Akt-PH was provided by Vesa Olkkonen (Minerva Foundation Institute for Medical Research). GFP-Tubby was given by Lawrence Shapiro (Columbia University, USA). GFP-Rac1 N17 and GFP-Rac1 V12 were gifts from Johan Peränen (University of Helsinki).

The hippocampal culture preparation was done according to [14, 15]. The dissection and dissociation of cells were carried out by the Neuroscience Center core facility (HiLIFE, University of Helsinki). Briefly, hippocampi were dissected from embryonic day 16–17 Wistar rat fetuses. Cells were dissociated in 0.05% papain and triturated in Ca^2+^ - and Mg^2+ ^^2^^+^^2^-free HBSS medium with 1 mM sodium pyruvate and 10 mM HEPES (pH 7.2). The obtained cells were then plated on 13 mm glass coverslips (VWR) (100,000 cells) or 13 mm high-precision glass coverslips for SIM (Marienfeld) (15,000 cells) coated with poly-L-Lysine (0.01 mg/ml, Sigma Aldrich) in a 24-well plate. The culturing of the cells was done in a Neurobasal medium (Invitrogen) supplemented with L-glutamine (Invitrogen), B-27 (Invitrogen), and primocin (InvivoGen) in humidified incubators at 37 °C and 5% carbon dioxide (CO2) with media refreshing twice a week. The transfection was performed at DIV14 by using Lipofectamine 2000 (Invitrogen) with 0.5 μg plasmid/well in 24-well plates, as described earlier [15]. Normally, the transfected cultures were fixed after 24 h of the transfection. For testing effects of F-actin depolymerization or PI3-kinase activity, cells were treated with 5 M Latrunculin B (Sigma-Aldrich) or 100 M PI3-kinase inhibitor LY294002 (Sigma-Aldrich) before fixation.

### Immunostaining

Hippocampal cultures were fixed with pre-warmed (37 °C) 4% paraformaldehyde (PFA) for 13 min at room temperature (RT), and after this, they were permeabilized using 0.2% TritonX-100 in PBS. Blocking was done for 30 min using 3% normal donkey serum and 0.5% bovine serum albumin (BSA) in PBS. The fixed cells were incubated with primary antibodies in a blocking solution for 1 h at RT, followed by incubation with secondary antibodies for 1 h. The coverslips with cells were mounted on glass slides by using Shandon Immu-Mount (Thermo Fisher Scientific, 9990402) or Prolong Gold (ThermoFisher, P10144). The primary antibodies used were rabbit anti-ABBA (1:400 [5], mouse anti-myc (9E10) (1:200, ThermoFisher, MA1-980), rabbit monoclonal anti-GAD65/67 (1:400, Abcam, AB 183999), guinea pig polyclonal antiserum against parvalbumin (1:500, Synaptic Systems, 195 004) and chicken polyclonal anti-GFAP (1:4000, Abcam, Ab4674). The secondary antibodies were anti-rabbit Alexa-488, anti-rabbit Alexa-647 (1:1000, ThermoFisher, A21206, A21446), anti-guinea pig Alexa-568 (1:1000, ThermoFisher, A11075), and anti-chicken Alexa-647 (1:1000, ThermoFisher, A-21449). F-actin was visualized using Alexa 488-conjugated phalloidin, Alexa 568-conjugated phalloidin, or Alexa 633-conjugated phalloidin (1:200, ThermoFisher, A12379, A12380, and A22284).

### Imaging

Confocal images were obtained using either a Zeiss LSM780 or LSM880 inverted confocal microscope. The fixed samples were imaged at room temperature, and the live cell imaging was performed in a chamber where the temperature was maintained at 37 °C and CO2 levels were 5%. The 63X 1.4 NA oil immersion objective was used for Z-stack imaging of each neuron. The step size was set to 0.2 μm. Cells or fields of view were selected randomly for imaging. The laser power and gain settings were adjusted to maximize the signal-to-noise ratio. The Fiji software was used to process the image files obtained from the imaging [16, 17].

SIM was performed as described earlier in [16]. The used imaging system was the DeltaVision OMX SR system (GE Healthcare Life Sciences) with a 60X  1.42 NA PlanApo N oil immersion objective. AcquireSR software was used for acquisition, and SoftWoRx for image reconstruction and alignment.

### Dendritic Spine Morphology and Density Analysis

NeuronStudio was used for the analysis of dendritic spine morphology and density. NeuronStudio is a software package designed for three-dimensional detection of dendritic spines from fluorescence microscopy images [18]. It also allows the classification of spines into different types based on their morphology. Images were re-numbered and ordered randomly before analysis, so that analysis was done blinded to the experimental condition. The Fiji software was used to convert Zeiss files with the z-stacks of 20–30 optical sections to the Tiff files. The images had a voxel size of 0.066 μm × 0.066 μm × 0.2 μm, and the EGFP or the mCherry channels were used for analyzing the dendritic spine morphology and density. After modeling the dendrite surface, protrusions with a minimum volume of 5 voxels (0.020 μm^3^), a length between 0.1 μm and 5 μm, and a maximal width of 3 μm were retained as spines. Following the default settings of the program and the empirical classification rule defined by [18], spines with a minimum head diameter of 0.35 μm and a minimum head vs. neck ratio of 1.1 were classified as mushroom spines. Non-mushroom spines with a minimum volume of 10 voxels (0.040 μm^3^) were classified as stubby spines. All other spines were considered thin. The spines detected and modeled by the software were carefully verified and corrected manually. The wrongly labeled structures were removed or corrected, and the spines that were not detected by the software were added and classified. Measurements obtained by NeuronStudio were transferred to a spreadsheet application (MS Excel) for further analysis.

### Co-localization Analysis

The co-localization analysis between GFP and mCherry-tagged constructs was done on the maximum intensity projections obtained from 3D Z-stacks. After making a maximum intensity projection, the GIMP software was used to outline the region of interest of the image for co-localization analysis. GIMP is an open-source drawing and annotation software (https://www.gimp.org/). The co-localization analysis was carried out with an EzColocalization Plugin in the Fiji software by following the procedure created, used, and reported in previous research [11, 19, 20]. Pearson’s correlation coefficient (PCC) value was determined for each image to measure the co-occurrence of two-channel signals. The range of PCC values is from 1 to −1, indicating a strong co-localization and a strong anti-co-localization, respectively. PCC = 0 reflects no correlation between the two signals.

### Cluster Analysis

Already for our previous research [11], we developed and created a new cluster analysis method and programmed a new R language script named "Neural image brightness cluster analysis script" [21, 22] to identify and analyze the emergence of brightness clusters in the given neural images (input images), motivated by the previous research and open source algorithm components [23, 24] among others R language libraries magrittr, tidyverse, imager, BiocManager, locfit, magick, spatstat, EBImage and ggplot2. That script is openly available at the following GitHub repository (https://github.com/laurilahti/neural-image-brightness-cluster-analysis-script/blob/main/neural-image-brightness-cluster-analysis-script-developed-by-lauri-lahti.R).

That script takes as inputs the maximum intensity projection Tiff images and their corresponding manually defined mask images, indicating a region of interest (ROI) to prevent background noise or crossing dendrites from being unintentionally included in the analysis. The script computes and outputs visualizations and exact numeric result files describing pixel regions that the script has identified in the input image file, matching the conditions defined by the script's adjustable parameter values about the desired brightness requirement (for example, the value 0.4 as the minimum brightness threshold value). The numeric result files describe, among others, the number of identified brightness regions per image and the area of each of these brightness regions.

While the script "Neural image brightness cluster analysis script" can produce and has already produced useful results [11], we noticed that a certain challenge is that the script relies on the global thresholding value (i.e., the emergence of brightness clusters is based on using the same minimum brightness threshold value for all parts of the image, for example 0.4). However, in a typical set of neural images (input images) acquired via confocal imaging, the lighting conditions and composition (such as exposure, dynamics, focus, positioning, distance, and overlap of objects) can vary a lot.

Therefore, for our current research, based on the script "Neural image brightness cluster analysis script", we developed and created a new alternative cluster analysis method and programmed a new alternative R language script version named "Neural image brightness cluster analysis script with adaptive thresholding" relying on the moving rectangular window and thresholding offset from the averaged value [25]. That new alternative script version is openly available at the following GitHub repository (https://github.com/laurilahti/neural-image-brightness-cluster-analysis-script/blob/main/neural-image-brightness-cluster-analysis-script-with-adaptive-thresholding-developed-by-lauri-lahti.R).

In our current research, the cluster analysis results have been generated specifically with this new adaptive thresholding script version, whereas in our previous research [11], they have been generated specifically with the original global thresholding script version.

Our new adaptive thresholding script version differs essentially from our original global thresholding script version, so that now the identified clusters may have reached varying brightness levels, and there is no longer a requirement that they have reached the same minimum brightness level in all parts of the image. Instead, now, when using the new adaptive thresholding script version, the reason for the cluster becoming identified relies essentially on having some sufficiently significant local brightness changes occurring in that part of the image (based on the moving rectangular window and thresholding offset from the averaged value).

The development of our new cluster analysis method and its results indicate that it is useful to carry out experimentation with a wide range of alternative desired brightness requirements to address the needs and goals of the research, depending on, among others, the circumstances of the experimental setups and the properties of their corresponding neural images. Then, based on human evaluation of their performance, it is practical to select the most promising and suitable desired brightness requirements for further steps of analysis.

Anyway, to ensure good comparability and generalizability of the results, we consider it recommendable to use the same brightness requirement when comparing and contrasting the images belonging to a certain shared experimental setup. In our current research, we have decided to use the value 10 as the half-width and the half-height of the moving rectangular window for adaptive thresholding.

Both the new adaptive thresholding script version and the original global thresholding script version support identification of brightness clusters based on agglomerating separate but relatively closely positioned bright pixels by using supplementary smoothed versions of input image files to identify brightness regions (this approach is motivated by, among others [23, 24].

To generate these supplementary smoothed versions of input image files, the script implements Gaussian blurring (i.e., smoothing) with desired adjustable parameter values of the size of "the brush" of the Gaussian kernel and the value of the sigma (σ) for "the brush" of the Gaussian kernel.

Based on our initial experimenting with various alternative parameter values and human evaluation of their performance we have decided to generate the cluster analysis results of our current research as well as the cluster analysis results of our previously reported research [11] by using systematically the value 51 as the size of "the brush" of the Gaussian kernel and the value 0.8 as the value of the sigma (σ) for "the brush" of the Gaussian kernel.

Further detailed description of our new adaptive thresholding script version and our original global thresholding script version, and their usage as well as defining and interpreting parameter values is openly available at the following GitHub repository guidance page (https://github.com/laurilahti/neural-image-brightness-cluster-analysis-script). Further details motivating the script development and its initial version are also described in the manuscript [21].

Despite using now the new adaptive thresholding script version, we still carried out in our current research similar general analysis steps as in our previous research [11] in which we used the original global thresholding script version, so that from the original 3D image stack data files, the maximum intensity projection tiff images were created by using in Fiji software the option "Image—> Stacks—> Z project", selecting all slices and using the projection type "Max Intensity", done separately for each image channel of the original 3D image stack data file.

To define the region of interest (ROI) for a neural image, one-pixel-wide white curves on black background (named "skeleton curve image") were manually drawn along the desired dendrite's branches by using a new online drawing tool that we have developed to enable and support better understanding and modeling of the human interpretation and decision making concerning neural images (or more broadly biomedical imaging data) and their analysis process [26].

The width of these skeleton curves was then widened 95 pixels (5 µm) to both sides of the curve by using in Gimp software option "Select—> Grow… (Grow selection)". This resulted in getting 191-pixel-wide "observation paths" that were saved as a black-and-white mask image in which the white color indicates all the pixels to be included in the further analysis of the maximum intensity projection tiff image. The length of the ROI for the desired dendrite was computed from the "skeleton curve image" by using in Fiji software option "Analyze—> Skeleton—> Analyze Skeleton (2D/3D)."

When implementing the cluster analysis, we used a carefully designed protocol to ensure non-biased analysis results. So that biologically meaningful results can be interpreted from the cluster analysis output files by human evaluation, it is necessary to run our scripts in a certain systematic order transparently (i.e., non-blindly) to enable mapping and linking correctly each set of cluster analysis output files to their corresponding experimental setups and neural image input files. Anyway, as is typical for computer programs in general, we programmed our scripts purposefully so that with respect to all the neural images recorded in different experimental setups, the mathematical logic of computation steps remains the same for each neural image file to ensure, in a non-biased way, the comparability and generalizability of computation outputs between groups and in groups.

When carrying out human evaluation concerning the computation outputs of the cluster analysis—such as to select the desired brightness requirements, to define the region of interest (ROI) for a neural image and to assess and verify a sufficient quality of the script's performance—we did this with a sincere aim to emphasize non-biased handling of the data in respect to different experimental setups. We supported this aim in human evaluation by, among others, interpreting the brightness patterns and defining the regions of interest (ROI) for neural images based on randomized sets of samples and hiding the groupings of experimental setups by using randomized file naming.

### Analyzing the Membrane vs Diffuse Protein Ratio for GFP-Tubby

The quantification of fluorescence intensity was done using single confocal focal planes in the middle (in the z direction) of the measured dendrites. We first draw lines along the sides presenting the plasma membrane, and in the middle of a dendrite, presenting diffuse protein using Fiji software. Measurements were averaged to get one average value for the membrane and one for diffuse protein, and the ratio was calculated using these average values.

### Statistical Analyses

Statistics were made in GraphPad Prism (GraphPad Software Inc.). We used either the two-sample t-test or two-way ANOVA for parametric data and the Kruskal–Wallis test or the Mann–Whitney U test for non-parametric data. The tests used are specified for each analysis in the Figure legends.

### Figures

Microscope images were processed in Fiji, Imaris (Oxford Instruments), and Photoshop (Adobe). Graphs were done in Prism (GraphPad Software Inc.). The final layout of figures was done in Inkscape.

## Results

### ABBA Is Highly Expressed in Parvalbumin-Positive Inhibitory Neurons and Radial Glia

We first tested the expression of ABBA in different mouse brain areas at different ages using Western blotting utilizing a previously published anti-ABBA antibody [1, 5] and compared ABBA-band intensity to the total protein (Fig. 1a-c). We compared expressions in different brain areas—cortex, hippocampus, and cerebellum, at two different ages, postnatal days 11 and 265 (P11 and P265) (Fig. 1a). In addition, we tested whether ABBA is expressed in our E16.5-derived rat hippocampal cultures at 14 days in vitro (DIV14). This comparison showed that at P11, ABBA expression was similar in all tested brain areas. At P265, expression was higher in the cerebellum compared to the cortex and hippocampus (Fig. 1a). ABBA was also expressed in hippocampal cultures (Fig. 1a). Next, we analyzed ABBA expression in each brain area from different ages, having two or three separate mice for each age (Fig. 1b). A comparison of relative ABBA expression against total protein revealed that after P11, expression in the cerebellum increases while expression in the cortex and hippocampus remains similar throughout life (Fig. 1c).

Next, we elucidated in which cells ABBA is expressed in basal conditions in the brain at different developmental stages. The Allen Brain Map transcriptomics explorer shows low expression of ABBA in almost all cell types in the brain. Inhibitory GABAergic neurons show higher expression of ABBA than excitatory glutamatergic neurons. Furthermore, some types of astrocytes and oligodendrocytes show relatively high expression of ABBA when compared to neuronal cells. We stained ABBA in sagittal mouse brain slices (Fig. 1d). At early development at P11, ABBA was expressed throughout the brain, often showing a pattern of sparse labeling. An exception to sparse labeling was a clear group of parvalbumin-positive cells in the area called the superior olivary complex (SOC) (Fig. 1e). At the cerebellum, ABBA showed high expression, especially in Purkinje cells and radial glial cells at both P11 and P250 (Fig. 1f). We stained Purkinje cells with anti-parvalbumin (PV) antibody and radial glial cells with anti-Glial Fibrillary Acidic Protein (GFAP) antibody. At later time points, ABBA and PV expression in Purkinje cells changed to be selective (Fig. 1f shows an area with prominent ABBA and PV expression). This selective expression for PV in aging Purkinje cells has also been observed before [27]. There were clear areas with Parvalbumin and ABBA-positive Purkinje cells and areas where these proteins were not expressed in Purkinje cells. Expression of ABBA in radial glial cells was less clear due to overall staining in the Purkinje cell dendrites. Additionally, ABBA was expressed in some, but not all, stellate cells labeled by anti-PV antibody staining.

In the hippocampus, ABBA showed a sparse labeling pattern, similar to the pattern seen with the *in-situ* hybridization technique utilized in the Allen Brain Atlas (mouse gene *Mtss1l,* Supplementary Fig. 2). Some ABBA-positive cells, but not all, expressed parvalbumin, suggesting that they were inhibitory neurons (Fig. 2a). In the cerebellum, ABBA was co-expressed with GFAP in radial glial cells. In the hippocampus, GFAP staining identifies astrocytes. We wanted to test whether ABBA is co-expressed with GFAP also in the hippocampus, and therefore, we co-stained slices with anti-GFAP antibody (Fig. 2a). However, ABBA expression was very low in GFAP-positive astrocytes. We also compared Thy1-promoter-regulated GFP expression with ABBA staining (Fig. 2b). Thy1-promoter-regulated GFP expression is mainly detected in pyramidal neurons in CA1-CA3 layers and granule cells in the dentate gyrus. ABBA mRNA expression was previously shown to be increased upon exercise and neuronal activity in granule cells in the dentate gyrus [2]. In basal conditions, expression was reported to be low [2], and our antibody staining confirmed this result (Fig. 2b). It is interesting that in the *in-situ* hybridization image provided by Allen Brain Atlas, there seems to be low ABBA mRNA expression in granule cells, but with ABBA antibody staining, these cells show no expression (Supplementary Fig. 2b, c), suggesting that maybe ABBA mRNA is expressed, but it is not translated to protein. While ABBA total expression in granule cells is low, local subcellular expression of ABBA may be enough to facilitate an increase in spine density. Using confocal imaging, we detected a few ABBA-positive clusters along granule cell dendrites (Fig. 2b, Dentate gyrus). While it was difficult to see from maximum projection whether ABBA-positive clusters were inside the dendrites, we created a 3D-co-localization channel for Thy-GFP and ABBA-antibody staining using Imaris software to visualize only those ABBA clusters that co-localize with GFP (Fig. 2b Zoom-ins). Co-localization channel showed only a few ABBA clusters inside the dendrites of granule cells. These clusters may serve as sites for spine initiation, but altogether, this analysis shows that ABBA protein expression without neuronal activation is below the level of immunofluorescence detection.

ABBA sparse staining pattern with equal distances from each other resembled the pattern seen with microglia; thus, we co-stained brain slices with anti-Iba1 antibody, the marker used to identify microglia in the brain. Similar to Thy1-GFP neurons, it seemed that ABBA was not expressed in microglia. With higher magnification (Fig. 2b, Dentate gyrus), we detected some ABBA clusters co-localizing with Iba1 staining, so we created a similar co-localization channel for Iba1- and ABBA-staining as we did for GFP and ABBA. Only very few ABBA clusters seemed to be inside the microglia (Fig. 2b, Zoom-ins), so we concluded that the ABBA protein expression in both granule neurons and at microglia is under the level of detection.

### Bicuculline and BDNF Treatments Increase the Expression of ABBA

ABBA expression was shown to be increased upon exercise [2]. As exercise increased the activity of granule cells in the dentate gyrus [2], we tested how bicuculline treatment affects ABBA expression. Bicuculline inhibits inhibitory neurons, thus activating excitatory neurons. Earlier, we showed that bicuculline treatment enhanced Gas7-clustering on the plasma membrane in excitatory pyramidal neurons in organotypic hippocampal slices [11]. Considering the potential role of ABBA in spine initiation, we also analyzed the expression of other identified spine initiation factors, SrGAP3 [28] and Gas7 [11].

Chatzi et al. demonstrated that physical exercise, which increased ABBA mRNA expression, was associated with elevated granule cell activity and BDNF expression in the dentate gyrus of the hippocampus [2]. Furthermore, BDNF treatment of primary neuron cultures increased ABBA expression [2]. Thus, in addition to bicuculline treatment, we tested whether we could detect expression changes upon BDNF treatment (Fig. 2c).

Slices were treated with bicuculline for 1 h, then incubated in their original media without the drug for 4 h, 1 day, or 2 days before being processed for Western blotting. For 4-h and 1-day BDNF treatment, slices were kept in BDNF for the whole time. Instead, for 2-day BDNF treatment, the slices were incubated first with BDNF for 1 day, and after this, BDNF-containing media were replaced with fresh media without BDNF. ABBA expression was significantly increased after 1 day of bicuculline treatment (Fig. 2c). Similarly, expression of SrGAP3 was significantly increased upon bicuculline treatment at 1-day follow-up. Although BDNF treatment produced a similar trend toward increased expression, the variability was higher, and statistical significance was not achieved.

Based on average expression levels, 4-h treatments with bicuculline or BDNF were insufficient to induce significant changes, and in 2 days, expression levels had generally started to decline (Fig. 2c). As we measure change from all cells and an increase in expression is expected to occur only in a subset of cells, it is understandable that detected changes are relatively small.

### ABBA Localizes to the Edges of Membrane Protrusions, Suggesting a Role in Cell Migration and Spinogenesis

**Fig. 3:**
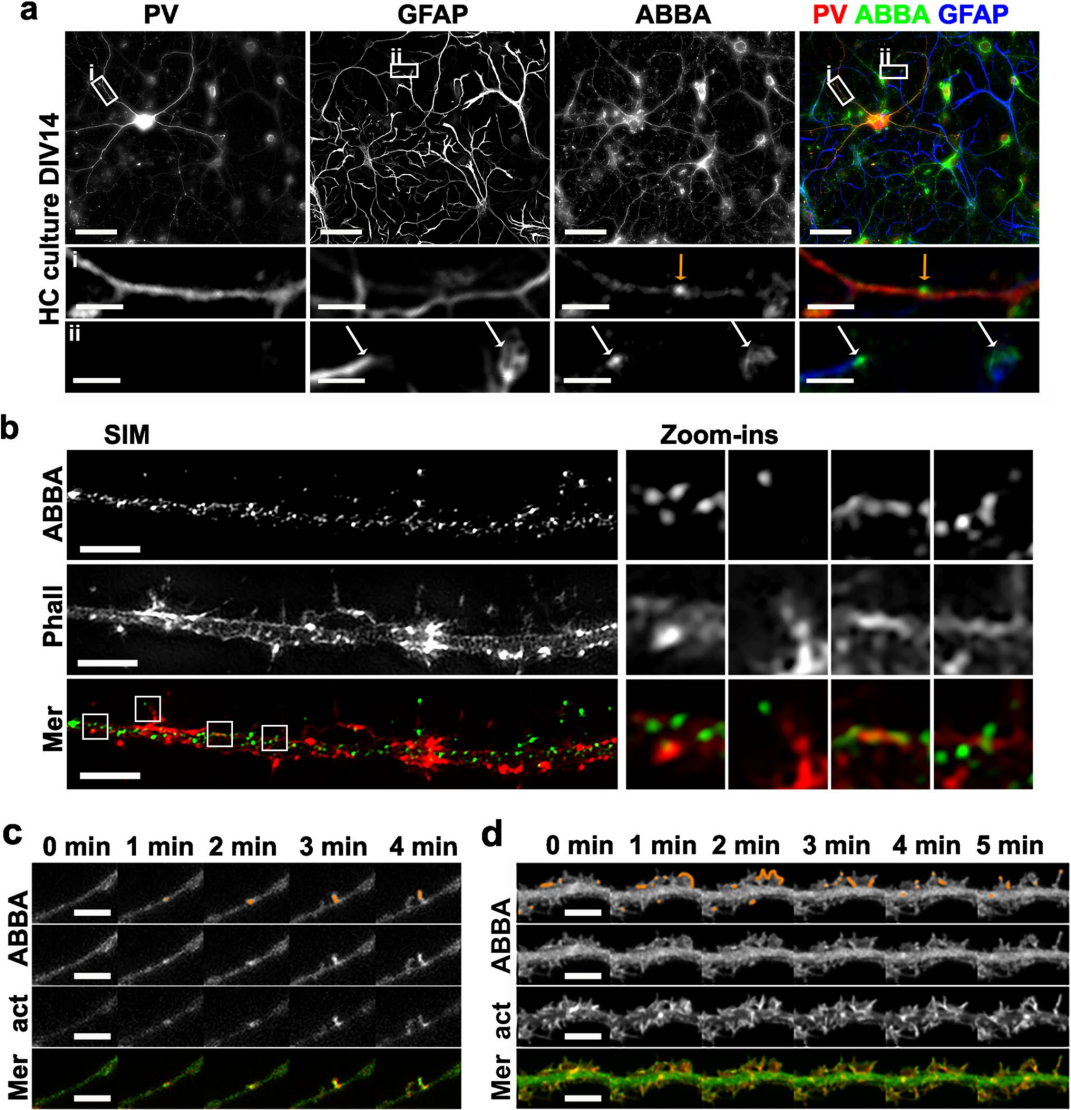
Expression and subcellular localization of ABBA in the hippocampal neuronal cultures. **a** DIV14 rat hippocampal (HC) neuronal cultures were stained with anti-ABBA, anti-PV, and anti-GFAP antibodies. The lower panels show zoomed-in images of i) one dendrite of the PV-positive neuron and ii) ends of astrocyte branches. White arrows mark ABBA-rich ends of astrocyte branches, and orange arrows mark potential spine initiation sites with ABBA-staining. Scale bars, 50 μm (upper panel) and 5 μm (lower panels). **b** DIV14 hippocampal neuronal cultures were stained with anti-ABBA antibodies and phalloidin to visualize filamentous actin. The images were acquired with a SIM super-resolution imaging system. Zoomed-in images from left to right show ABBA-localization compared to filamentous actin, the tip of a dendritic filopodium, and at two potential spine initiation sites on the dendrite. Scale bars, 2 μm. **c** Time frames of a segment of a hippocampal neuron transfected with GFP-ABBA (ABBA) and mCherry-actin (act). ABBA occasionally clusters at the spine initiation sites where new filopodia appear at later time points. ABBA localization is highlighted in the images in the first row with orange coloring. Scale bars, 5 μm. See also Video 1. **d** Time frames of a segment of hippocampal neuron transfected with GFP-ABBA and mCherry-actin show that new filopodia (last frame, on the right) can also form from lamellipodial structures. ABBA localization is highlighted in the images in the first row with orange coloring. Scale bars, 5 μm. See also Video 2

We detected the ABBA expression in hippocampal cultures by Western blotting (Fig. 1a). To elucidate which cells express ABBA in rat hippocampal cultures, we stained cultures similar to the brain slices with PV and GFAP antibodies to distinguish inhibitory parvalbumin-positive neurons from excitatory neurons and labelled astrocytes with GFAP. There were very few parvalbumin-expressing neurons in the hippocampal cultures. ABBA was expressed in all neurons, and the selectivity between inhibitory and excitatory neurons seen in brain slices was absent (Fig. 3a). In inhibitory neurons, ABBA was enriched in a few protrusions along their dendrites (Fig. 3a, upper zoom-in panel). Astrocytes showed very low ABBA expression (Fig. 3a). However, with higher magnification, ABBA expression was detected at the ends of astrocyte branches (Fig. 3a, lower zoom-in panel), as shown before [1].

We have previously studied spine initiation factors MIM and Gas7 in excitatory neurons [10, 11]. To be able to compare ABBA to those factors, we wanted to carry more detailed characterization of ABBA localization and function in excitatory neurons. Furthermore, in hippocampal cultures, there was no clear difference in ABBA expression between PV-positive and PV-negative neurons (Fig. 3a).

We increased the imaging resolution by utilizing structured illumination microscopy (SIM) imaging. Hippocampal cultures were stained with anti-ABBA antibody and phalloidin, which stain filamentous actin (Fig. 3b). At this resolution, ABBA co-localized with actin filaments only occasionally (Fig. 3b, first zoom-in). Endogenous ABBA localized to the tips of protrusions, especially in dendritic filopodia (Fig. 3b, second zoom-in). It was also found from potential spine initiation sites on the dendrite (Fig. 3b, last zoom-ins).

To follow the dynamics of ABBA, we transfected cultured neurons with GFP-ABBA and RFP-Lifeact to visualize filamentous actin (Fig. 3c, d). We were interested in seeing whether ABBA can localize to spine initiation sites. These are sites on dendrites that serve as starting points for new filopodia or spines. Indeed, we could occasionally identify ABBA clustering sites, which were followed by the growth of small filopodia, similarly to our earlier findings for MIM [10] or Gas7 [11] (Fig. 3c, Video 1). However, the likelihood of finding these sites was lower than for MIM or Gas7, suggesting that this may not be a typical or only function for ABBA. Indeed, ABBA was often localized on the edge of lamellipodial structures on dendrites (Fig. 3d, Video 2). These lamellipodial structures are partially overexpression artifacts, as they are seldom seen in control cells. However, in this experimental setup, lamellipodia formation reveals the different mechanism utilized by ABBA compared to other spine initiation factors.

### ABBA Co-localizes with a PI(4,5)P2 Marker, and ABBA Clustering Is Enhanced by PI3-Kinase Inhibition

Previously, we showed that PI(4,5)P2/PI(3,4,5)P3 signaling is an important upstream regulator for the membrane recruitment of I- and F-BAR domain-containing proteins MIM and Gas7 [10, 11]. Furthermore, Gas7 localization depended on phosphoinositide 3-kinase (PI3-kinase) activity, indicating that it preferred PI(3,4,5)P3 over PI(4,5)P2. It has also been shown earlier that the ABBA I-BAR domain interacts with PI(4,5)P2-rich membranes and deforms them into tubular structures [1]. Thus, we wanted to test whether ABBA co-localizes with PI(4,5)P2 and/or PI(3,45)P3 markers.

**Fig. 4:**
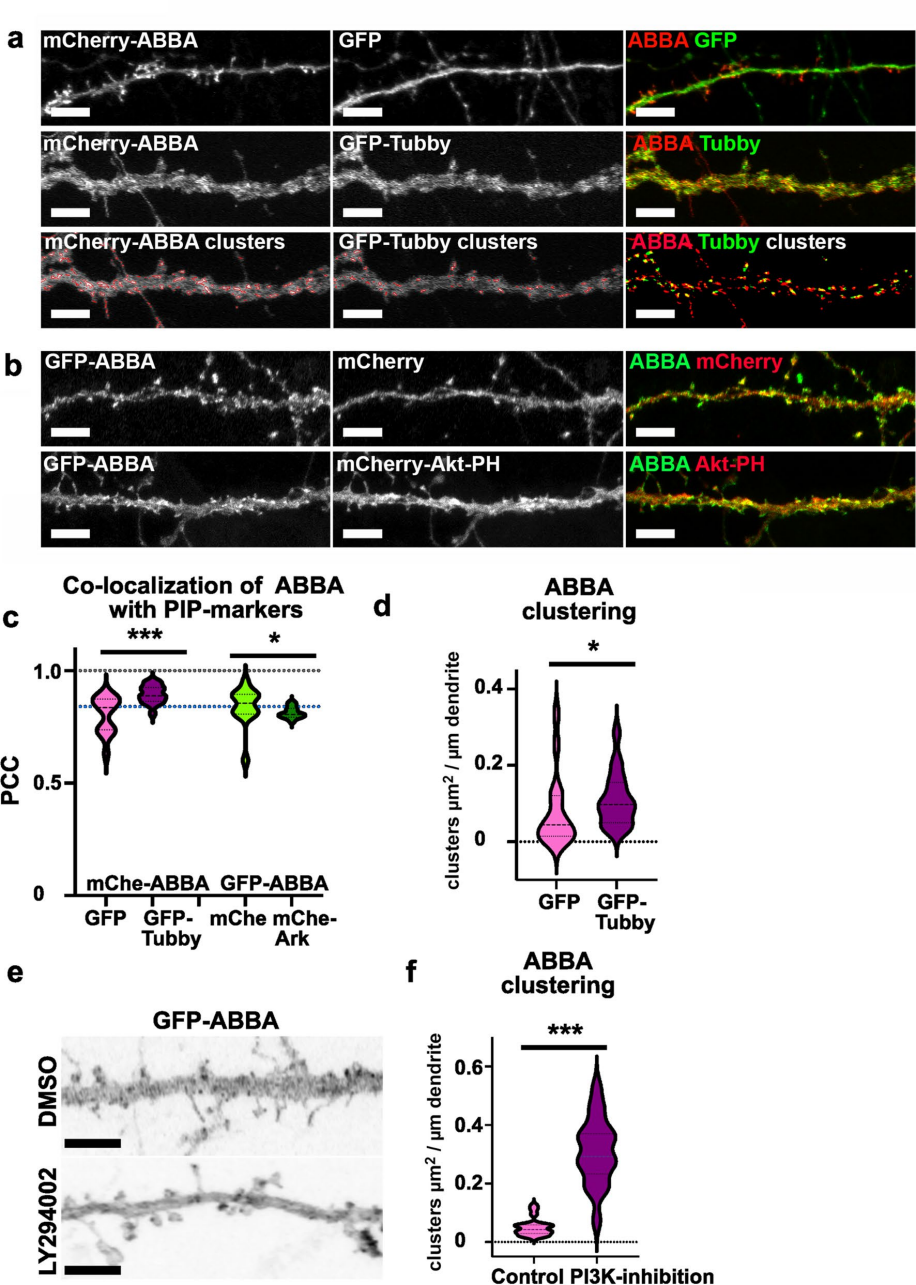
ABBA co-localizes with PI(4,5)P2-marker and ABBA clustering is enhanced with PI3-kinase inhibition. **a** The uppermost panel shows a dendritic segment of primary hippocampal neurons expressing mCherry-ABBA and GFP, and the middle panel shows a dendrite expressing mCherry-ABBA and GFP-Tubby. The lowest panel shows visually cluster analysis results with selected values used as the thresholding offset from the averaged value. Results of ABBA clustering are shown in d. Scale bars, 5 μm. **b** The upper panel shows a dendritic segment of primary hippocampal neurons expressing GFP-ABBA and mCherry. The lower panel shows the dendritic segment expressing GFP-ABBA and mCherry-Akt-PH. Scale bars, 5 μm. **c** Quantification of colocalization of mCherry-ABBA with GFP or GFP-Tubby and GFP-ABBA with mCherry or mCherry-Akt-PH. To determine the co-occurrence of the two signals, Pearson’s correlation coefficient (PCC) values were calculated for each image. PCC values ranged from −1 (indicating a strong anti-colocalization) to + 1 (indicating a strong colocalization). The value of 1 referred to 100% colocalization. PCC values of 0 show no correlation between the two signals. The results are represented here as a mean ± SEM, *p < 0.05, ***p < 0.001, determined by the Mann–Whitney U test. mCherry-ABBA with GFP (n = 15 neurons); or GFP-Tubby (n = 18 neurons, three experiments), GFP-ABBA with mCherry (n = 15 neurons); or mCherry-Akt-PH (n = 14 neurons, two experiments). The PCC value for expressing mCherry-ABBA with GFP = 0.805 ± 0.021; or GFP-Tubby = 0.895 ± 0.009, GFP-ABBA with mCherry = 0.844 ± 0.021; or mCherry-Akt-PH = 0.813 ± 0.007. The violin plot presents the median and interquartile range (25th and 75th percentiles). **d** The quantification of mCherry-ABBA clustering in primary hippocampal neurons transfected with GFP or GFP-Tubby, showing clustering μm^2^ per dendrite length μm. The data were pooled from three independent experiments (GFP: n = 16 neurons, Mean ± SEM 0.078 ± 0.024; GFP-Tubby: n = 18 neurons, 0.110 ± 0.016). *p < 0.05, determined by the Mann–Whitney U test. **e** The dendritic segments of GFP-ABBA expressing neurons that were treated with DMSO control (upper panel) or 100 μM LY294002 (PI3-kinase inhibitor) for 10 min. Scale bars, 5 μm.  **f** The quantification of clusters of GFP-ABBA in GFP-ABBA-expressing neurons treated with DMSO or 100 μM LY294002. ***p < 0.001, determined by unpaired t-test, four experiments, DMSO: n = 20; LY294002: n = 19. GFP-ABBA clustering μm^2^ per dendrite length μm for DMSO-treated cells was mean ± SEM 0.0505 ± 0.007, and for LY294002-treated neurons was 0.310 ± 0.026. The violin plot presents the median and interquartile range (25th and 75th percentiles)

We first tested the co-localization of mCherry-ABBA with a marker binding PI(4,5)P2 (GFP-Tubby). We compared ABBA-Tubby co-localization to the co-localization of mCherry-ABBA with free GFP. As ABBA-Tubby co-localization value was greater than the value with the diffuse control, we considered this result as a positive indication of co-localization (Fig. 4a, c). Co-localization of GFP-ABBA with PI(3,4,5)P3 was tested using the mCherry-Akt-PH construct, which binds PI(3,4)P2 and PI(3,4,5)P3, and co-localization was compared to free mCherry (Fig. 4b). As the ABBA-Akt-PH co-localization value was significantly lower than that with diffuse control, we considered this to indicate less co-localization than would be expected from randomly localized proteins (Fig. 4c). These results suggest that ABBA prefers PI(4,5)P2-rich plasma membrane areas over PI(3,4,5)P3.

When highly overexpressed, PIP markers can also compete with proteins binding the same PIPs out of location [29–31]. Therefore, we analyzed ABBA clustering in the presence of Tubby expression. We further developed our previously utilized cluster analysis by using a dynamic thresholding method, which detects clusters based on the local brightness contrast rather than just overall intensity [21, 22, 25]. Cluster analysis results are shown visually in Fig. 4A for a neuron co-expressing mCherry-ABBA and GFP-Tubby. The cluster analysis showed that ABBA clustered more in the presence of GFP-Tubby, confirming that Tubby expression did not compete ABBA out from the PIP2-rich areas on the plasma membrane (Fig. 4d).

We further tested the effect of PI3-kinase inhibition on ABBA clustering. PI3-kinase inhibition depleted Gas7 from the plasma membrane [11] but increased MIM clustering [32]. PI3-kinase inhibition with 100 µM LY294002 for a 10-min treatment increased the total clustering of ABBA compared to control cells (Fig. 4e, f). This result suggests that PI(3,4,5)P3 is not required for ABBA´s localization or clustering. When PI(3,4,5)P3 production decreases, level of PI(4,5)P2 increases [31, 33]. The most plausible explanation for the increased clustering of ABBA upon PI3-kinase inhibition is that elevated PI(4,5)P2 levels enhance ABBA clustering.

To test whether PI(4,5)P2 levels increase on the plasma membrane upon PI3-kinase inhibition, we repeated the same PI3-kinase inhibition assay for neurons expressing GFP-Tubby (Supplementary Fig. 3). The analysis of Tubby levels at the plasma membrane vs. in the middle of dendrites from the middle focal plane of a confocal stack revealed that Tubby localized more on the membrane after the PI3-kinase inhibition, suggesting that there is more PI(4,5)P2 on the plasma membrane after PI3-kinase inhibition.

### ABBA Co-localizes with Inactive and Active Rac1

**Fig. 5:**
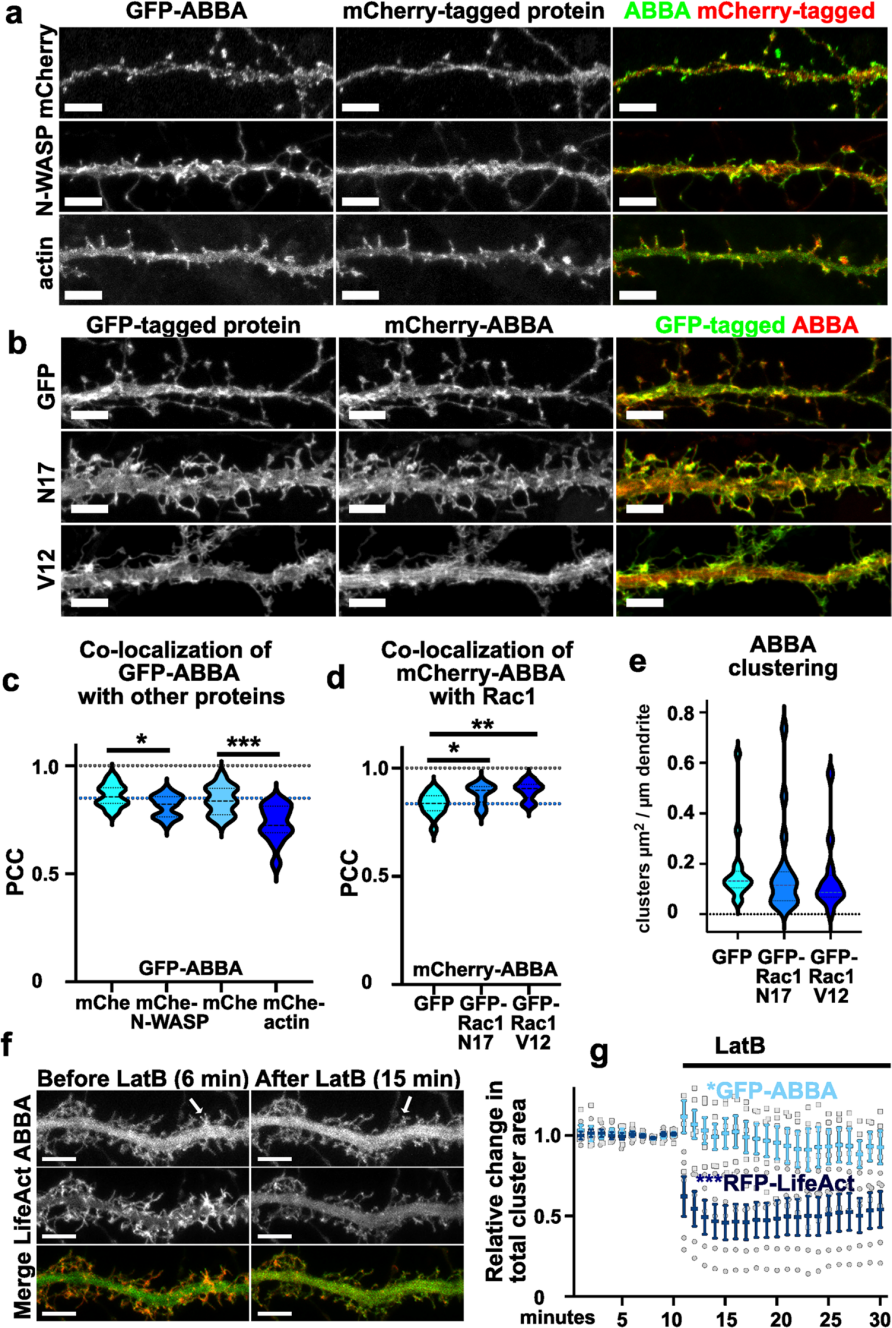
Overexpressed ABBA co-localizes with Rac1 but not with N-WASP or actin. **a** The uppermost panel shows a dendritic segment of a primary hippocampal neuron expressing GFP-ABBA and mCherry. The middle panel shows a dendritic segment of a primary hippocampal neuron expressing GFP-ABBA and N-WASP-mCherry. The lowest panel shows a dendritic segment of a neuron transfected with GFP-ABBA and mCherry-Actin. Scale bars, 5 µm. **b** The uppermost panel shows a dendritic segment of a primary hippocampal neuron expressing GFP and mCherry-ABBA. The middle panel shows a dendritic segment of a primary hippocampal neuron expressing GFP-Rac1-N17 and mCherry-ABBA. The lowest panel shows a dendritic segment of a neuron transfected with GFP-Rac1-V12 and mCherry-ABBA. Scale bars, 5 µm. **c** Quantification of colocalization of GFP-ABBA and NWASP-mCherry or mCherry-actin in primary hippocampal neurons. N(neurons) for GFP-ABBA with mCherry (n = 15); or N-WASP-mCherry (n = 16) or GFP-ABBA with mCherry (n = 16); or mCherry-actin (n = 27). Data is pooled from three experiments. The violin plot presents the median and interquartile range (25th and 75th percentiles). There is a significant negative change in co-localization values for both N-WASP-mCherry and mCherry-actin compared to free mCherry. *p < 0.05, ***p < 0.001 determined by t-test. PCC values of GFP-ABBA with mCherry = 0.863 ± 0.013, N-WASP-mCherry = 0.814 ± 0.012; mCherry = 0.840 ± 0.016; or mCherry-Actin = 0.734 ± 0.016 (mean ± SEM). **d** Quantification of colocalization in primary hippocampal neurons transfected with mCherry-ABBA and either GFP, GFP-Rac1 N17, or GFP-Rac1 V12. Data were pooled from four independent experiments, with the following sample sizes (neurons): GFP (n = 17), GFP-Rac1 N17 (n = 18), and GFP-Rac1 V12 (n = 14). The violin plot displays the median and interquartile range (25th and 75th percentiles). Significant differences were detected in the colocalization of mCherry-ABBA with GFP, GFP-Rac1 N17, and GFP-Rac1 V12. Statistical significance *p < 0.05 and **p < 0.01 was determined by one-way ANOVA, using the Kruskal–Wallis test. Pearson correlation coefficient (PCC) values for mCherry-ABBA colocalization were as follows: GFP = 0.835 ± 0.012, GFP-Rac1 N17 = 0.880 ± 0.011, and GFP-Rac1 V12 = 0.897 ± 0.011 (mean ± SEM). **e** The quantification of mCherry-ABBA clusters in neurons where ABBA was co-expressed with either GFP, GFP-Rac1 N17, or GFP-Rac1 V12, showing clustering μm^2^ per dendrite length μm. Data is pooled from four experiments. N of analyzed neurons GFP = 17; Rac1 N17 = 18, and Rac1 12 V = 13. The violin plot presents the median and interquartile range (25th and 75th percentiles). **f** Timeframes from time-lapse video of a dendritic segment of a primary hippocampal neuron transfected with RFP-LifeAct and GFP-ABBA before and after 5 μM latrunculin B treatment. Video was recorded 10 min before the Latrunculin B treatment and 20 min after adding Latrunculin B. Time stamps are shown as time after the start of recording. Scale bars, 5 µm. See also Video 3. **g** Prior to latrunculin B (LatB) treatment, RFP-LifeAct showed clustering. However, upon the addition of LatB, RFP-LifeAct clusters dissolved. In contrast, GFP-ABBA clusters remained largely unaffected by LatB treatment. Data were obtained from five cells across three independent experiments. Results are presented as mean ± SEM of relative values, normalized to the average total cluster area measured in four frames prior to LatB treatment. Statistical significance was determined after LatB treatment, using the Wilcoxon Signed Rank Test, with *p < 0.05 for GFP-ABBA and ***p < 0.001 for RFP-LifeAct, indicating significant differences from the hypothetical value of 1

Previously characterized spine initiation factors srGAP3, MIM, and Gas7 showed slight differences in actin co-localization and regulation [10, 11, 28, 34]. Neural Wiskott-Aldrich Syndrome protein (N-WASP) belongs to a family of actin nucleation-promoting factors that regulate actin-cytoskeleton network rearrangement by activating the actin-related protein 2/3 (Arp2/3) complex. Gas7 bound N-WASP directly via its WW-domain [11, 34] and MIM showed co-localization with N-WASP [10]. Thus, we wanted to test whether ABBA co-localizes with N-WASP or actin. We co-transfected primary hippocampal neurons with GFP-ABBA combined with mCherry, mCherry-N-WASP, or mCherry-actin (Fig. 5a).

In previous reports, ABBA has been shown to co-localize with Rac1 [1, 35]. This co-localization did not depend on the activity status of Rac1, in other words, whether Rac1 bound GDP or GTP [1, 35]. We wanted to test whether this co-localization can be detected in neurons and transfected neurons with mCherry-ABBA combined with GFP, GFP-Rac1-N17 (inactive Rac1), or GFP-Rac1-V12 (active Rac1) (Fig. 5b). Rac1-N17 is a mutated construct that binds continuously to GDP, and V12 is a mutated Rac1, which binds continuously to GTP and is therefore continuously active.

In co-localization analysis, ABBA co-localization values for N-WASP or actin were significantly smaller compared to the diffuse protein (Fig. 5c). In contrast, co-localization values for both Rac1-constructs were significantly higher than co-localization with free GFP (Fig. 5d). No differences in co-localization were detected between inactive (N17) or active (V12) Rac1. Expression of the Rac1-constructs did not affect clustering of ABBA (Fig. 5e).

We tested whether actin polymerization is required for ABBA localization by transfecting neurons with RFP-LifeAct to visualize filamentous actin and GFP-ABBA to visualize ABBA localization. We recorded time-lapse videos 10 min before and 20 min after adding the Latrunculin B. Selected time-lapse frames show frames before Latrunculin B addition (6 min after starting the video recording, 4 min before adding Latrunculin B and 5 min after adding Latrunculin B, showing a timepoint 15 min after starting the video recording) (Fig. 5f, Video 3). Filamentous actin depolymerized upon latrunculin B treatment, resulting in a decrease in the total cluster area of LifeAct (Fig. 5g). Analysis of the total GFP-ABBA cluster area showed a slight, slow decrease during follow-up imaging (Fig. 5g). However, this decrease was very modest and did not follow the loss of actin filaments. From this experiment, we can conclude that filamentous actin was not required for ABBA clustering, as depolymerization of actin filaments with latrunculin B had only a modest effect on the clustering of ABBA. While actin polymerization was not required for ABBA clustering, it was required for the dynamics of ABBA. Video 3 shows that before latrunculin B treatment, GFP-ABBA localization changes from frame to frame, whereas dynamics freeze after adding latrunculin B.

### Overexpression of ABBA Enhances the Rate of Actin Depolymerization in the Presence of Latrunculin B

**Fig. 6:**
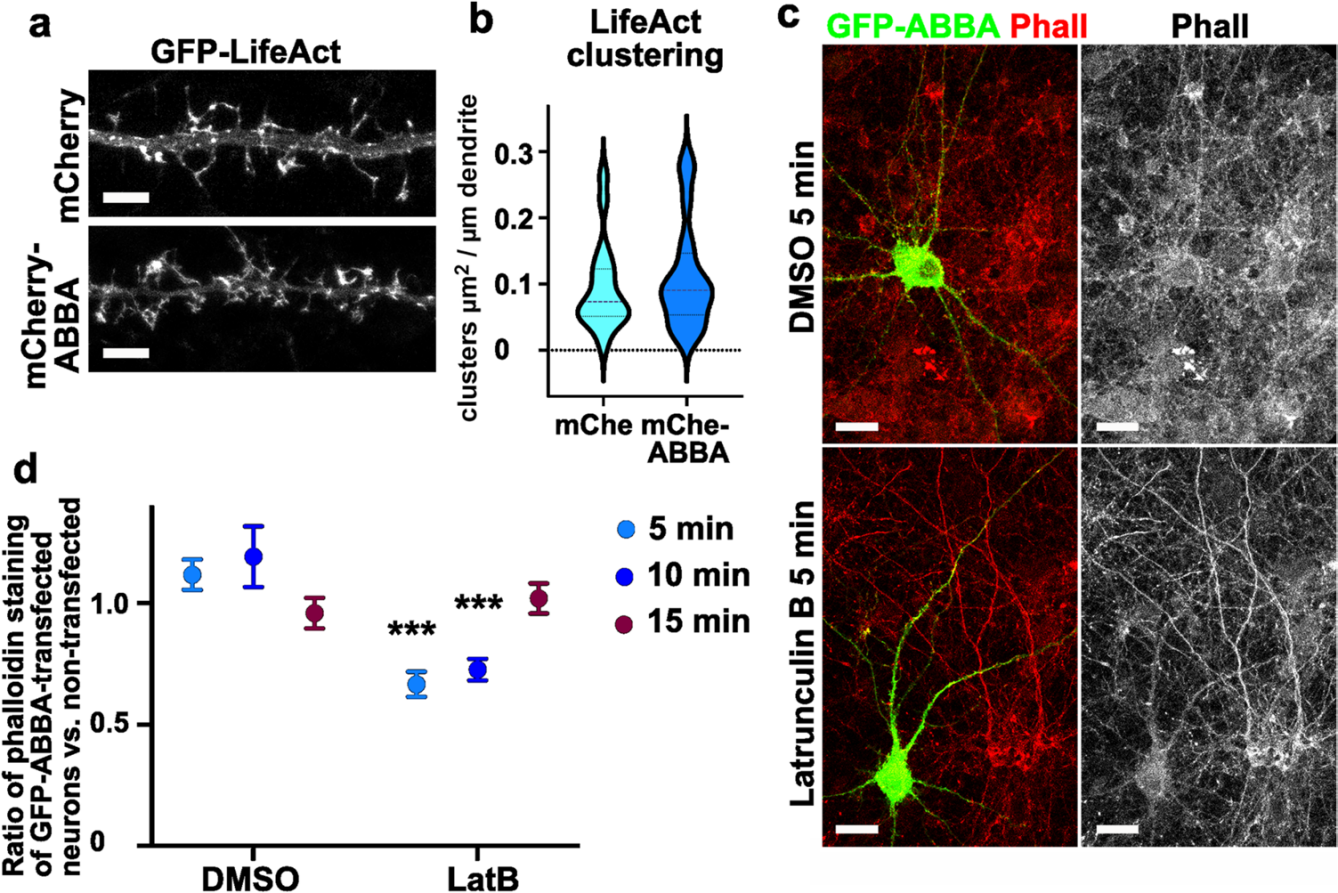
Overexpression of ABBA enhances the rate of actin depolymerization in the presence of latrunculin B. **a** The dendritic segment of a primary hippocampal neuron transfected with GFP-LifeAct, either with mCherry or mCherry-ABBA. Scale bars, 5 µm. **b** The quantification of clusters of GFP-LifeAct with either mCherry or mCherry-ABBA, showing clustering μm^2^ per dendrite length μm. Data is pooled from four experiments, mCherry = 19, mCherry-ABBA = 19. The violin plot presents the median and interquartile range (25th and 75th percentiles). The change is not significant. **c** The upper panel shows the dendritic segment of a primary hippocampal neuron transfected with GFP-ABBA, treated with DMSO and LatB treatment for 5 min, and immunostained with phalloidin. **d** The quantification was done by measuring the ratios of phalloidin staining of GFP-ABBA-transfected neurons vs. non-transfected neurons after treatment with DMSO and LatB for 5, 10, and 15 min. Data pooled from 3 experiments. DMSO 5 min: n = 24; DMSO 10 min: n = 16, and DMSO 15 min: n = 21. LatB 5 min: n = 22; LatB 10 min: n = 20; LatB 15 min: n = 20. There is a significant change in the groups of the treatment with DMSO and LatB after 5 and 10 min. ***p < 0.001 determined by two-way ANOVA with Sidak’s multiple comparison test. Ratios of phalloidin staining for cells treated with DMSO 5 min: 1.116 ± 0.06; DMSO 10 min: 1.190 ± 0.12; DMSO 15 min: 0.958 ± 0.06. Ratios of phalloidin staining for cells treated with LatB 5 min: 0.684 ± 0.05; LatB 10 min: 0.711 ± 0.04; LatB 15 min: 0.995 ± 0.05

In our previous study, Gas7 strongly increased actin clustering [11]. Therefore, we tested whether ABBA can also increase actin clustering, and we co-transfected hippocampal neurons with mCherry-ABBA and GFP-LifeAct and compared this to mCherry and GFP-LifeAct control cells (Fig. 6a). In contrast to Gas7, ABBA did not increase actin clustering (Fig. 6b).

To test whether ABBA affects the dynamics of F-actin, we treated cells with the actin monomer sequestering drug latrunculin B for 5, 10, and 15 min (Fig. 6c shows a 5-min treatment). We transfected primary hippocampal neurons with GFP-ABBA and stained cells with phalloidin to detect F-actin (Fig. 6c). Then, we analyzed phalloidin intensity in GFP-ABBA-expressing cells and compared this value to phalloidin intensity measured from non-transfected cells in the same image. If ABBA overexpression did not affect the amount of F-actin, the ratio should always be 1. In DMSO-treated cells, the ratio was approximately 1.0–1.2, suggesting that ABBA expression either increases or does not affect the amount of F-actin in neurons (Fig. 6d). However, when cells were treated with latrunculin B, which sequesters actin monomers and thus blocks actin polymerization, leading to depolymerization of F-actin, GFP-ABBA-expressing neurons exhibited lower phalloidin staining intensity than non-transfected cells (Fig. 6c). Intensity analysis measuring the ratio of phalloidin staining in ABBA-overexpressing cells vs. non-transfected cells confirmed this (Fig. 6d). After a 5-min 5 µM Latrunculin B treatment, the ratio of phalloidin staining in ABBA-expressing cells vs. non-transfected cells dropped to 0.7, suggesting that F-actin depolymerized faster in ABBA-overexpressing cells. We observed this same effect in the 10-min time point (ratio 0.7), but not anymore by the 15-min time point, where the ratio was back to 1 (Fig. 6d). This result suggests that ABBA´s overexpression enhanced the depolymerization of actin filaments.

### ABBA-Induced Increase in Spine Density Requires Arp2/3-Complex but Not Rac1 Activity

**Fig. 7:**
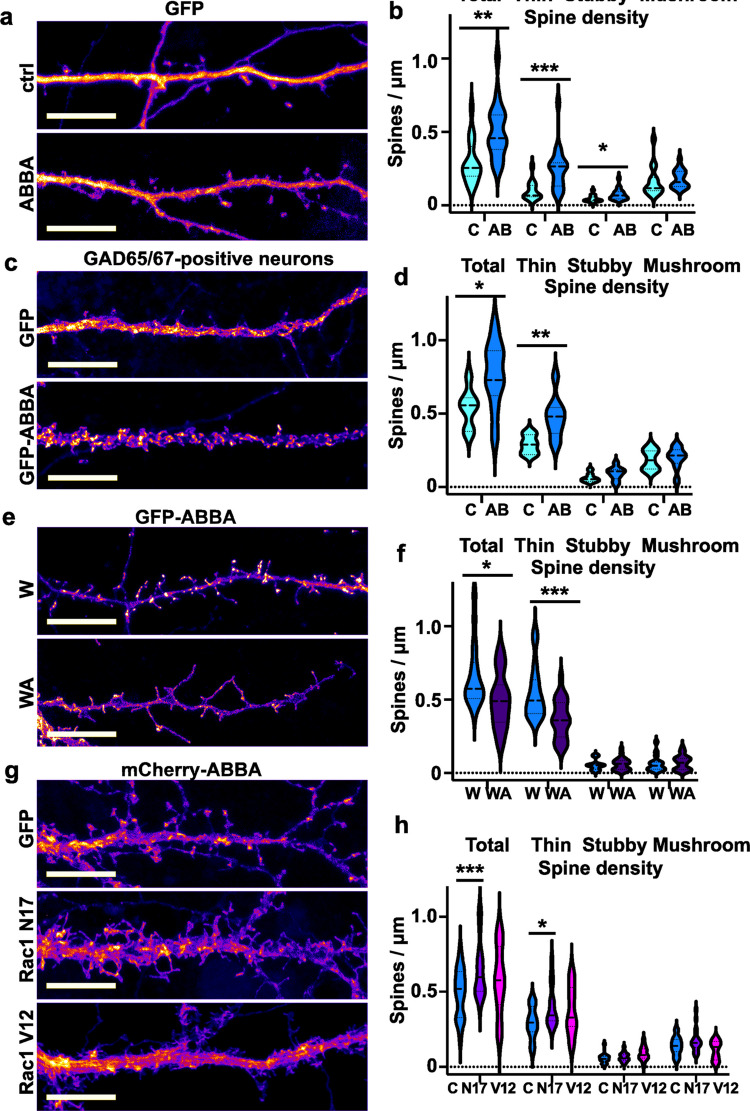
Overexpression of ABBA increases the spine density. **a** A representative dendritic segment of a hippocampal DIV15 neuron transfected with GFP and mCherry (control, upper panel), or GFP and mCherry-ABBA (lower panel). The scale bar is 10 μm. Pseudocolored with intensity-based Fire. **b** The quantification of dendritic spine density is calculated as the number of spines per m of a dendrite. The total spine density for control was 0.295 ± 0.039 and for mCherry-ABBA-overexpressing cells was 0.504 ± 0.043. The number of thin spines for control was 0.097 ± 0.016, and for mCherry-ABBA-overexpressing cells 0.251 ± 0.033. The stubby spine densities were 0.044 ± 0.006 for controls vs. 0.073 ± 0.009 for mCherry-ABBA. *p < 0.05, **p < 0.01 or ***p < 0.001 as determined by Mann–Whitney U test. Mushroom spines did not show significant results (control: n = 15 neurons; ABBA: n = 19 neurons). Data were pooled from 3 independent experiments. **c** A representative dendritic segment of a hippocampal DIV15 inhibitory neuron transfected with GFP (control, upper panel) or GFP-ABBA (lower panel), identified as inhibitory neurons with GAD65/67 antibody staining (See Supplementary Fig. 7). The scale bar is 10 μm. Pseudocolored with intensity-based Fire. **d** Quantification of dendritic spine density is calculated as the number of spines per m of a dendrite. The total spine density for control was 0.539 ± 0.050, and for GFP-ABBA overexpressing cells was 0.765 ± 0.075. The number of thin spines for control was 0.290 ± 0.024, and for GFP-ABBA-overexpressing cells was 0.472 ± 0.046. The stubby and mushroom spines did not show significant changes (control: n = 7 neurons; ABBA: n = 9 neurons). Data were pooled from 3 independent experiments. *p < 0.05 or **p < 0.01 as determined by t-test. **e** A representative dendritic segment of a hippocampal DIV15 neuron transfected with GFP-ABBA, together with either Scar W-myc (upper panel) or Scar WA-myc (lower panel). Myc-tag was immunostained with an anti-myc secondary Alexa-647 antibody (not shown). The scale bar is 10 μm. Pseudocolored with intensity-based Fire. **f** Quantification of dendritic spine density is calculated as the number of spines per m of a dendrite. The total spine density for GFP-ABBA with Scar W-myc was 0.660 ± 0.052, and for GFP-ABBA with Scar WA-myc overexpressing cells was 0.496 ± 0.039. The number of thin spines for GFP-ABBA with Scar W-myc: 0.544 ± 0.044; or Scar WA-myc: 0.365 ± 0.028. *p < 0.05 or ***p < 0.001 as determined by t-test. The stubby and mushroom spines did not show significant results (W: n = 17 neurons; WA: n = 23 neurons). Data were pooled from 4 independent experiments. **g** A representative dendritic segment of a hippocampal DIV15 neuron transfected with mCherry-ABBA with GFP (control, the uppermost panel), mCherry-ABBA with GFP-Rac1 N17 (middle panel), and mCherry-ABBA with GFP-Rac1 V12 (the lowest panel). The scale bar is 10 μm. Pseudocolored with intensity-based Fire. **h** Quantification of dendritic spine density is calculated as the number of spines per m of a dendrite. The total spine density of mCherry-ABBA for GFP: 0.490 ± 0.037; GFP-Rac1 N17: 0.645 ± 0.044; and GFP-Rac1 V12: 0.581 ± 0.066). The number of thin spines of mCherry ABBA with GFP: 0.293 ± 0.024; GFP-Rac1 N17: 0.400 ± 0.032; GFP-Rac1 V12: 0.375 ± 0.047. *p < 0.05 or ***p < 0.001 as determined by the two-way ANOVA. The stubby and mushroom spines did not show significant results (GFP: n = 19 neurons; Rac1 N17: n = 18 neurons; Rac1 V12: n = 12 neurons). Data were pooled from 4 independent experiments

Chatzi et al. demonstrated that overexpression of ABBA increased dendritic spine density in both in vitro hippocampal cultures and in vivo in mouse dentate gyrus granule cells [2]. To confirm these results, we repeated the experiment using rat hippocampal cultures. Indeed, ABBA´s overexpression increased the spine density (Fig. 7a, b). In morphological analysis, where we separated thin, mushroom, and stubby spines, the density of thin spines was particularly increased (Fig. 7b). Thus, we confirmed earlier results showing that increased ABBA expression has a positive effect on spine density.

As we showed that ABBA was mainly expressed in inhibitory neurons in the mouse hippocampus (Fig. 2a), we wanted to test how ABBA overexpression affects inhibitory neuron morphology in cultures. We transfected hippocampal cultures as before and then identified inhibitory neurons with anti-GAD65/67 antibody staining (See Supplementary Fig. 4). In GAD65/67-positive neurons, ABBA overexpression significantly increased total and thin spine density (Fig. 7c, d).

Both MIM [10] and Gas7 [11] induced spine initiation were dependent on Arp2/3-complex-dependent actin polymerization [1], and therefore, we tested whether Arp2/3-complex-mediated actin assembly is required for ABBA-induced increase in spine density (Fig. 7e, f). For this, we used the Scar WA-myc construct as we did previously for MIM [10] and Gas7 [11]. WA is a conserved sequence domain in proteins of the WASP family that binds to the Arp2/3 complex. Overexpression of this domain in cells disrupts the localization of the Arp2/3 complex, thereby inhibiting its normal functionality [13]. The Scar W-myc construct, which does not bind to the Arp2/3 complex, was used as a negative control [13]. Quantification of spine density in ABBA-GFP/WA-co-expressing cells demonstrated that Arp2/3-complex activity is critical for ABBA function in potentiating spine formation (Fig. 7e, f). Co-expression of the Scar-WA domain with ABBA reduced the ABBA-induced increase in spine density (Fig. 7e, f).

ABBA interacts with Rac1, and deletion of ABBA in C6-R cells markedly inhibited Rac1 activation and cell spreading [9]. The authors concluded that the interaction between ABBA and activated Rac1 is required for ABBA-promoted cell spreading [9]. Following this, we wanted to test whether Rac1 activity is required for an ABBA-induced increase in spine density. Thus, we co-expressed ABBA with inactive and active Rac1 constructs and compared the spine phenotype to ABBA expressed together with free GFP (Fig. 7G). Based on earlier results, we expected that the inactive Rac1 construct would diminish the ABBA-induced increase in spine density; however, the analysis showed the opposite result. Rac1-constructs, Rac1 N17 particularly, enhanced the increase in spine density (Fig. 7h). Rac1 V12 had a relatively strong phenotype, having many small filopodia or lamellipodia, which were maybe not always typical spines but were now counted as spines. Taken together, we can conclude that Rac1 activity was not necessary for ABBA-induced spine formation. Together with our co-localization results, we summarize that although ABBA and Rac1 co-localize, Rac1 activity is not required for ABBA-facilitated increase in spine density.

## Discussion

To better understand the role of ABBA in the brain, we investigated its age-, brain region, and cell type-specific expression in the mouse brain under basal and activity-dependent conditions. By identifying enriched ABBA expression in inhibitory parvalbumin-positive interneurons (Fig. 2a) and radial glia (Fig. 1f), our findings expand the cellular context of ABBA function beyond excitatory neurons. Using organotypic hippocampal slices, we found that bicuculline treatment increased ABBA expression (Fig. 2c). At the subcellular level, ABBA localized to the edges of membrane protrusions (Fig. 3b-d), suggesting functions in cell migration and spinogenesis. ABBA co-localized with PI(4,5)P2 (Fig. 4a,c), and inhibition of PI3-kinase increased the PI(4,5)P2 levels and clustering of ABBA on the plasma membrane (Fig. 4d, f, Supplementary Fig. 3). Previous reports have shown that ABBA binds Rac1, and we could detect co-localization of ABBA with both active and inactive Rac1 in neurons (Fig. 5b, d).

We showed that ABBA enhanced actin dynamics, particularly by enhancing actin depolymerization (Fig. 6c, d). Overexpression of ABBA in pyramidal excitatory and inhibitory neurons increased dendritic spine density (Fig. 7a-d). In hippocampal neurons, this effect required Arp2/3 complex activity (Fig. 7e, f). Rac1 activity was not required for the increased spine density, as the expression of inactive Rac1 further increased spine density in ABBA-expressing pyramidal neurons (Fig. 7g, h).

Our findings advance our understanding of ABBA’s role in neurodevelopmental processes and disorders by providing insight into ABBA expression, localization, and functional mechanisms. Huang et al. [3] identified an *MTSS2* mutation in individuals with syndromic intellectual disability and demonstrated its toxicity using a *Drosophila* model, suggesting a dominant-negative mechanism. However, the cellular mechanism and mammalian context of ABBA dysfunction remained unexplored. *Drosophila* is a powerful model to study genetic mutations, but *Drosophila* neurons do not exhibit similar dendritic spines as mammalian neurons, making it impossible to study effects on spine initiation in *Drosophila*. Therefore, our study brings new insights into whether the *MTSS2* mutation could affect spine initiation. Furthermore, *Drosophila* Missing-in-metastasis (*mim*) is a fly ortholog for both MIM and ABBA, and therefore, these genes cannot be studied separately [3]. Based on our results, we reason that defective spine initiation can play a role in *MTSS2*-linked syndromic intellectual disability, but this might not be the only cause for observed symptoms, because ABBA appears to be a multifunctional protein. In general, ABBA coordinates the processes occurring at the interface of the plasma membrane and the actin cytoskeleton. This coordination facilitates the formation of diverse protrusions in various cell types.

While ABBA expression was low or even absent in some cells where it has been shown to have a fundamental function, a small amount of protein with strictly regulated expression in both time and space may be sufficient for certain functions. For example, low, strictly localized, and tightly regulated expression of ABBA in dentate gyrus granule cells would be suitable for activity-induced dendritic spine formation demonstrated by [2]. While the discovery of ABBA as a neuron activity-regulated protein associated with spine formation was highly interesting, the molecular mechanisms behind this effect were not fully characterized. Our study addresses this gap by demonstrating, through live-cell imaging, that ABBA facilitates spine initiation by clustering on the plasma membrane before a new filopodium appears (Fig. 3c). We originally hypothesized that ABBA initiates spines similar to its close homologue MIM, which induced purely finger-like protrusions on neural dendrites [10]. However, our video analysis revealed that ABBA localized not only to small focal points, typical for filopodia formation, on the plasma membrane, but also more broadly on the edge of lamellipodial structures (Fig. 3d). Furthermore, the likelihood of finding spine initiation sites was lower than for MIM [10] or Gas7 [11], suggesting that this may not be a typical or only function for ABBA. Compared to MIM, ABBA appears to be a more general facilitator of protrusion formation, from dendritic filopodia to lamellipodial structures. Likely, interactions with other protein(s) at the plasma membrane will define whether protrusion will be in the form of dendritic filopodia or broader lamellipodia-like protrusions. While this type of high ABBA overexpression might never occur, this type of in vitro experiment helps to understand the molecular characteristics of ABBA, especially when results are compared to earlier results with other spine initiation factors.

For MIM-induced spine initiation, we proposed that MIM induces PI(4,5)P2 clustering, which recruits a PI(4,5)P2-responsive activator of Arp2/3 complex, such as N-WASP, to the spine initiation site [10]. Following this same pathway, we detected co-localization of ABBA and PI(4,5)P2 (Fig. 4a, c) using the PI(4,5)P2 marker Tubby. ABBA´s binding to PI(4,5)P2 has been shown earlier [1], so in neurons, we mainly wanted to see whether ABBA localizes on PI(4,5)P2-rich areas and whether it co-localizes more with PI(4,5)P2 or PI(3,4,5)P3. Results indicated that ABBA co-localized with PI(4,5)P2 marker Tubby. We cannot exclude the possibility that ABBA and Tubby interact with each other resulting in co-localization and increased clustering of ABBA, but taken together earlier information on ABBA´s binding to PI(4,5)P2 [1] and our results that PI3-kinase inhibition increased clustering of ABBA and membrane localization of Tubby separately, we think it is safe to conclude that PI(4,5)P2 directs ABBA on the plasma membrane.

**Fig. 8:**
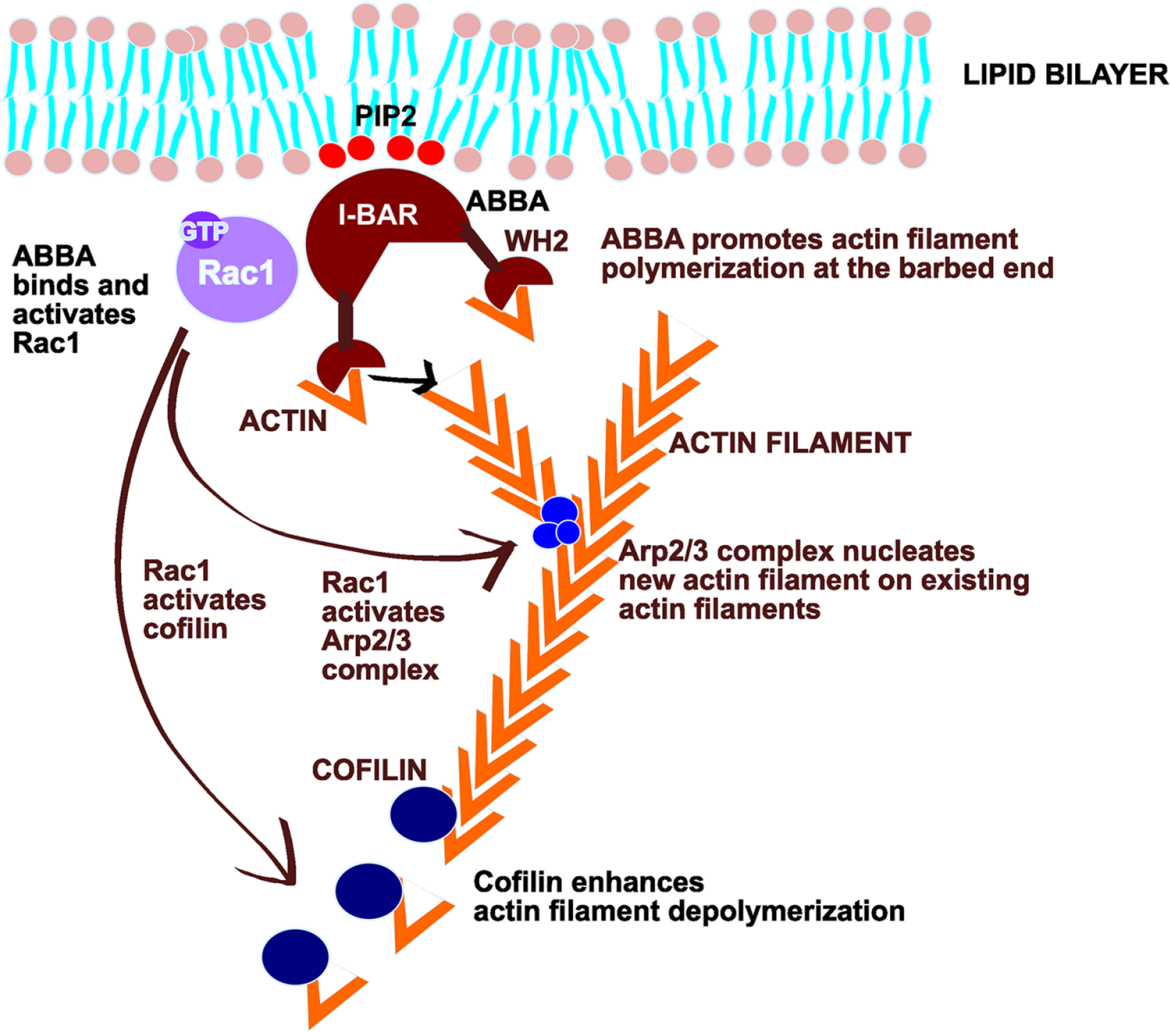
Working model. Based on our results and data published previously, we created a working model for how ABBA could regulate actin dynamics. This is a simplified model and does not try to present all existing data. We propose that PI(4,5)P2 recruits ABBA to the plasma membrane. In the illustration, we draw ABBA as a dimer when it can bend the plasma membrane with its I-BAR domain. ABBA also contains the WH2 domain binding ATP-actin monomers, which can be added to the barbed ends of actin filaments. However, it is unclear whether ABBA facilitates polymerization. ABBA binds Rac1 in both GTP and GDP forms, and in other cell types, ABBA activates Rac1. Rac1 regulates numerous actin-binding proteins, here presented cofilin and Arp2/3 complex. Activation is indirect via other proteins, and arrows visualize signaling pathways. Cofilin enhances actin depolymerization, whereas Arp2/3 complex facilitates actin nucleation and polymerization, inducing a branched actin filament network.

For actin regulation, we first tested whether ABBA and N-WASP co-localize, but they did not show any co-localization (Fig. 5a, c). Arp2/3 complex activity was required for the ABBA-induced increase in spine density (Fig. 7e, f). However, ABBA did not show any strong actin phenotype under control conditions, and therefore it is possible that it does not directly increase the Arp2/3 complex activity, but Arp2/3 complex activity is generally necessary for spine initiation. In contrast to polymerization, we showed that ABBA enhanced actin filament depolymerization in the Latrunculin B assay (Fig. 6c, d). In Latrunculin B-treated cells, Latrunculin B sequesters actin monomers, leading to depolymerization of actin filaments because there are no monomers to be added to the barbed ends. It is unclear how ABBA enhances this depolymerization, but earlier studies showed that ABBA promotes Rac1 activation [9], and we showed that ABBA co-localized with Rac1-constructs in primary neurons. Rac1 can activate many other proteins, including Arp2/3 complex and cofilins. Cofilins enhance depolymerization by severing actin filaments and accelerating the depolymerization (Fig. 8).

Rac1 activity was not required for ABBA-induced increase in spine density (Fig. 7g, h). This was surprising at first glance, but when taking a closer look at actin regulation in spine initiation, the result is not so surprising. SrGAP3, known spine initiation factor, has a Rac1 GAP domain and “switches off” Rac1 signaling. However, in the context of dendritic filopodia initiation, the SrGAP3 iF-BAR domain was as effective as the full-length protein in inducing filopodia [28]. Thus, Rac1-regulation was not needed [28]; see also [34]. Based on current literature, Rac1 seems not to be necessary for spine initiation, but it is necessary for spine head growth and stabilization [34]. This might explain that while ABBA can activate Rac1 in different cellular contexts [9], this activation is not necessary in spine initiation.

### Limitations of the Study

This study was inspired by previous ABBA results. We were especially excited about ABBA´s potential role as an activity-induced spine initiation factor, and we thought to characterize the mechanistic characteristics for ABBA, as we had done for another spine initiation factors, MIM [10] and Gas7 [11]. We did not plan to do any knock-down studies because these were well done in [2]. Chatzi et al. 2019 investigated granule cells in the dentate gyrus and observed a defect in exercise-induced spine formation in ABBA knock-down neurons three days after exercise. The increase in spine density was associated with a corresponding increase in functional synaptic input in exercise-activated control cells but not in ABBA knockdown cells [2]. EPSC amplitudes in paired recordings were unaffected by shRNA-ABBA expression in granule cells that were not activated by exercise, indicating that knockdown of ABBA had no effect unless the granule cell was exercise-activated [2]. As we now know that there is no ABBA expressed in granule cells under basal conditions (Fig. 2) and exercise increased ABBA expression 100-fold [2], it is plausible that at basal conditions ABBA knockdown does not affect cells because there is no ABBA expression to knockdown. We think that our overexpression studies can be considered to model the situation after the exercise.

A perfect mechanistic study would have been to study mechanistic details in the same experimental setting as Chatzi et al., but this was not feasible for us. The closest feasible experimental model was excitatory neurons in primary hippocampal cultures. In cultures, we classify pyramidal neurons and granule cells together as “excitatory neurons” based on their morphology. While the detected low expression of ABBA in vitro excitatory cells would have allowed us to decrease ABBA expression, we did not do it, because we did not expect to see any phenotype with ABBA knockdown in vitro cultures, where we cannot reproduce exercise-induced effects. Thus, our experiments provide mechanistic insights into how ABBA can facilitate spine formation and how mechanisms differ from its close homologue MIM. At the same time, we don’t provide evidence to demonstrate that ABBA is important for spine initiation in these cells.

During the study, the results of the analysis of ABBA expression in various cell types in the brain revealed that instead of pyramidal neurons or granule cells, we should focus on inhibitory neurons under basal conditions, because ABBA is highly expressed in those cells. In vitro primary neuron cultures never revealed this difference in expression, showing their limited differentiation. Working with inhibitory neurons in cultures is challenging because there are only a few inhibitory neurons per 100 000 cells, whereas there are numerous excitatory neurons in these same cultures. While expression studies showed that we should have focused more on inhibitory cells, we think that mechanistic studies in excitatory neurons are still reasonable. First, ABBA was required for spine formation when these cells were activated in vivo. Second, mechanisms inducing spines in different types of neurons are expected to be relatively similar. Therefore, studies in excitatory neurons bring us insights into mechanisms utilized also in inhibitory neurons.

In the future, ABBA´s role in inhibitory neurons should be revealed, possibly by knocking it down, especially in parvalbumin-positive inhibitory neurons in vivo. It would also be interesting to see how the mutated ABBA construct would behave in interneurons and how it would affect their functionality.

## Concluding Remarks

This study revealed that ABBA is highly expressed in parvalbumin-positive inhibitory neurons and radial glia in the brain, highlighting a previously underappreciated cell-type specificity for this protein in neurodevelopment. Based on our results, we would expect that a disease-related variant of *MTSS2* would primarily affect these cells that express ABBA at high levels. Expression pattern changes during development and aging (Fig. 1a-c), which may explain the different phenotypes of the ABBA mutation at different ages.

We focused mechanistic studies on primary hippocampal neurons and spine initiation. We revealed insights into how ABBA is regulated by PI(4,5)P2 binding and how ABBA affects the actin cytoskeleton dynamics. We also showed that ABBA differs from its close homologue MIM in the regulation of the spine initiation.

## Supplementary Information

Below is the link to the electronic supplementary material.

ESM 1 (PNG 863 KB)

ESM 1 (TIF 15.4 MB)

ESM 2 (PNG 1.82 MB)

ESM 2 (TIF 18.7 MB)

ESM 3 (PNG 1.04 MB)

ESM 3 (TIF 15.6 MB)

ESM 3 (PNG 443 KB)

ESM 4 (TIF 8.04 MB) 

ESM 5 (PDF 2.71 MB)

ESM 6 (MP4 5.44 MB)

ESM 7 (MP4 4.56 MB)

ESM 8 (MP4 3.62 MB)

## Data Availability

The datasets generated and analyzed during the current study are publicly available in the repository Dryad. DOI: 10.5061/dryad.ttdz08m8g.

## References

[1] Saarikangas J, Hakanen J, Mattila PK, Grumet M, Salminen M, Lappalainen P (2008) ABBA regulates plasma-membrane and actin dynamics to promote radial glia extension. J Cell Sci 121(9):1444–1454. 10.1242/jcs.027466 18413296 10.1242/jcs.027466

[2] Chatzi C, Zhang Y, Hendricks WD, Chen Y, Schnell E, Goodman RH, Westbrook GL (2019) Exercise-induced enhancement of synaptic function triggered by the inverse BAR protein, Mtss1L. Elife. 10.7554/eLife.45920 31232686 10.7554/eLife.45920 PMC6609409

[3] Huang Y, Lemire G, Briere LC, Liu F, Wessels MW, Wang X, Osmond M, Kanca O et al. (2022) The recurrent de novo c.2011C>T missense variant in MTSS2 causes syndromic intellectual disability. Am J Hum Genet 109(10):1923–1931. 10.1016/j.ajhg.2022.08.011 36067766 10.1016/j.ajhg.2022.08.011 PMC9606386

[4] Corona-Rivera JR, Zenteno JC, Ordonez-Labastida V, Cruz-Cruz JP, Cortes-Pastrana RC, Pena-Padilla C, Bobadilla-Morales L, Corona-Rivera A et al. (2023) MTSS2-related neurodevelopmental disorder: further delineation of the phenotype. Eur J Med Genet 66(10):104826. 10.1016/j.ejmg.2023.104826 37657631 10.1016/j.ejmg.2023.104826

[5] Carabalona A, Kallo H, Gonzalez M, Andriichuk L, Elomaa E, Molinari F, Fragkou C, Lappalainen P et al. (2024) Identification of novel microcephaly-linked protein ABBA that mediates cortical progenitor cell division and corticogenesis through NEDD9-RhoA. eLife Sciences Publications, Ltd. 10.7554/elife.92748.1 10.7554/eLife.92748 PMC12286603 40698928

[6] Saarikangas J, Zhao H, Lappalainen P (2010) Regulation of the actin cytoskeleton-plasma membrane interplay by phosphoinositides. Physiol Rev 90(1):259–289. 10.1152/physrev.00036.2009 20086078 10.1152/physrev.00036.2009

[7] Hertzog M, van Heijenoort C, Didry D, Gaudier M, Coutant J, Gigant B, Didelot G, Preat T et al (2004) The beta-thymosin/WH2 domain; structural basis for the switch from inhibition to promotion of actin assembly. Cell 117(5):611–623. 10.1016/s0092-8674(04)00403-9 15163409 10.1016/s0092-8674(04)00403-9

[8] Dayel MJ, Mullins RD (2004) Activation of Arp2/3 complex: addition of the first subunit of the new filament by a WASP protein triggers rapid ATP hydrolysis on Arp2. PLoS Biol 2(4):E91. 10.1371/journal.pbio.0020091 15094799 10.1371/journal.pbio.0020091 PMC387265

[9] Zeng XC, Luo X, Wang SX, Zhan X (2013) Fibronectin-mediated cell spreading requires ABBA-Rac1 signaling. J Cell Biochem 114(4):773–781. 10.1002/jcb.24415 23060091 10.1002/jcb.24415

[10] Saarikangas J, Kourdougli N, Senju Y, Chazal G, Segerstråle M, Minkeviciene R, Kuurne J, Pieta GL et al. (2015) MIM-Induced Membrane Bending Promotes Dendritic Spine Initiation. Dev Cell 33(6):644–659. 10.1016/j.devcel.2015.04.014 26051541 10.1016/j.devcel.2015.04.014

[11] Khanal P, Boskovic Z, Lahti L, Ghimire A, Minkeviciene R, Opazo P, Hotulainen P (2023) Gas7 is a novel dendritic spine initiation factor. eNeuro 10(4):ENEURO.0344-0322. 10.1523/eneuro.0344-22.2023 10.1523/ENEURO.0344-22.2023 PMC10114493 36963834

[12] Riedl J, Crevenna AH, Kessenbrock K, Yu JH, Neukirchen D, Bista M, Bradke F, Jenne D et al. (2008) Lifeact: a versatile marker to visualize F-actin. Nat Methods 5(7):605–607. 10.1038/nmeth.1220 18536722 10.1038/nmeth.1220 PMC2814344

[13] Machesky LM, Insall RH (1998) Scar1 and the related Wiskott-Aldrich syndrome protein, WASP, regulate the actin cytoskeleton through the Arp2/3 complex. Curr Biol 8(25):1347–1356. 10.1016/s0960-9822(98)00015-3 9889097 10.1016/s0960-9822(98)00015-3

[14] Abouelezz A, Stefen H, Segerstråle M, Micinski D, Minkeviciene R, Lahti L, Hardeman EC, Gunning PW et al. (2020) Tropomyosin Tpm3.1 is required to maintain the structure and function of the axon initial segment. iScience 23(5):101053. 10.1016/j.isci.2020.101053 32344377 10.1016/j.isci.2020.101053 PMC7186529

[15] Hotulainen P, Llano O, Smirnov S, Tanhuanpää K, Faix J, Rivera C, Lappalainen P (2009) Defining mechanisms of actin polymerization and depolymerization during dendritic spine morphogenesis. J Cell Biol 185(2):323–339. 10.1083/jcb.200809046 19380880 10.1083/jcb.200809046 PMC2700375

[16] Schindelin J, Arganda-Carreras I, Frise E, Kaynig V, Longair M, Pietzsch T, Preibisch S, Rueden C et al. (2012) Fiji: an open-source platform for biological-image analysis. Nat Methods 9(7):676–682. 10.1038/nmeth.2019 22743772 10.1038/nmeth.2019 PMC3855844

[17] Schindelin J, Rueden CT, Hiner MC, Eliceiri KW (2015) The ImageJ ecosystem: an open platform for biomedical image analysis. Mol Reprod Dev 82(7–8):518–529. 10.1002/mrd.22489 26153368 10.1002/mrd.22489 PMC5428984

[18] Rodriguez A, Ehlenberger DB, Dickstein DL, Hof PR, Wearne SL (2008) Automated three-dimensional detection and shape classification of dendritic spines from fluorescence microscopy images. PLoS ONE 3(4):e1997. 10.1371/journal.pone.0001997 18431482 10.1371/journal.pone.0001997 PMC2292261

[19] Lahti L (2021) Data analysis supplement to the research article Abouelezz, Stefen, Segerstråle, Micinski, Minkeviciene, Lahti, Hardeman, Gunning, Hoogenraad, Taira, Fath, & Hotulainen (2020), "Tropomyosin Tpm3.1 Is Required to Maintain the Structure and Function of the Axon Initial Segment". http://urn.fi/URN:NBN:fi:aalto-202109109107. Accessed 18 Nov 2025 10.1016/j.isci.2020.101053 PMC7186529 32344377

[20] Micinski D, Lahti L, Abouelezz A (2022) Measuring Properties of the Membrane Periodic Skeleton of the Axon Initial Segment using 3D-Structured Illumination Microscopy (3D-SIM). J Vis Exp (180). 10.3791/63327 10.3791/63327 35225255

[21] Lahti L (2022a) Developing ethical and transparent artificial intelligence algorithms to support decision making in healthcare based on brain research and personal care events of patients. http://urn.fi/URN:NBN:fi:aalto-202207154400. Accessed 18 Nov 2025

[22] Lahti L (2022b) Neural image brightness cluster analysis script. https://github.com/laurilahti/neural-image-brightness-cluster-analysis-script. Accessed 18 Nov 2025

[23] Hagler DJ Jr, Saygin AP, Sereno MI (2006) Smoothing and cluster thresholding for cortical surface-based group analysis of fMRI data. Neuroimage 33(4):1093–1103. 10.1016/j.neuroimage.2006.07.036 17011792 10.1016/j.neuroimage.2006.07.036 PMC1785301

[24] Holmes S, Huber W (2022) Modern statistics for modern biology. https://web.stanford.edu/class/bios221/book/. Accessed 18 Nov 2025

[25] Lahti L (2025a) Neural image brightness cluster analysis script with adaptive thresholding. https://github.com/laurilahti/neural-image-brightness-cluster-analysis-script/blob/main/neural-image-brightness-cluster-analysis-script-with-adaptive-thresholding-developed-by-lauri-lahti.R. Accessed 18 Nov 2025

[26] Lahti L (2025b) Development of neural image analysis method. https://tukiapu.fi/research/development-of-neural-image-analysis-method-by-lauri-lahti-2025. Accessed 18 Nov 2025

[27] Brandenburg C, Smith LA, Kilander MBC, Bridi MS, Lin Y-C, Huang S, Blatt GJ (2021) Parvalbumin subtypes of cerebellar Purkinje cells contribute to differential intrinsic firing properties. Mol Cell Neurosci 115:103650. 10.1016/j.mcn.2021.103650 34197921 10.1016/j.mcn.2021.103650

[28] Carlson BR, Lloyd KE, Kruszewski A, Kim I-H, Rodriguiz RM, Heindel C, Faytell M, Dudek SM et al. (2011) *WRP/srGAP3* facilitates the initiation of spine development by an inverse F-BAR domain, and its loss impairs long-term memory. J Neurosci 31(7):2447–2460. 10.1523/jneurosci.4433-10.2011 21325512 10.1523/JNEUROSCI.4433-10.2011 PMC3541779

[29] Cernikova L, Faso C, Hehl AB (2020) Phosphoinositide-binding proteins mark, shape and functionally modulate highly-diverged endocytic compartments in the parasitic protist *Giardia lamblia*. PLoS Pathog 16(2):e1008317. 10.1371/journal.ppat.1008317 32092130 10.1371/journal.ppat.1008317 PMC7058353

[30] Simon MLA, Platre MP, Assil S, Van Wijk R, Chen WY, Chory J, Dreux M, Munnik T et al (2014) A multi-colour/multi-affinity marker set to visualize phosphoinositide dynamics in Arabidopsis. Plant J 77(2):322–337. 10.1111/tpj.12358 24147788 10.1111/tpj.12358 PMC3981938

[31] Tariq K, Luikart BW (2021) Striking a balance: PIP2 and PIP3 signaling in neuronal health and disease. Exp Neuroprotective Ther 1(2). 10.37349/ent.2021.00008 10.37349/ent.2021.00008 PMC8797975 35098253

[32] Khanal P (2023) Molecular regulation of the dendritic spine initiation. Helsingin yliopisto, Helsinki. http://hdl.handle.net/10138/563362. Accessed 18 Nov 2025

[33] Ivanova A, Atakpa-Adaji P, Rao S, Marti-Solano M, Taylor CW (2024) Dual regulation of IP(3) receptors by IP(3) and PIP(2) controls the transition from local to global Ca(2+) signals. Mol Cell 84(20):3997-4015.e3997. 10.1016/j.molcel.2024.09.009 39366376 10.1016/j.molcel.2024.09.009

[34] Khanal P, Hotulainen P (2021) Dendritic spine initiation in brain development, learning and diseases and impact of BAR-domain proteins. Cells. 10.3390/cells10092392 34572042 10.3390/cells10092392 PMC8468246

[35] Zheng D, Niu S, Yu D, Zhan XH, Zeng X, Cui B, Chen Y, Yoon J et al. (2010) *Abba* promotes PDGF-mediated membrane ruffling through activation of the small GTPase Rac1. Biochem Biophys Res Commun 401(4):527–532. 10.1016/j.bbrc.2010.09.087 20875796 10.1016/j.bbrc.2010.09.087 PMC2980902

