# Supplementary figures and images for: Expression, Subcellular Localization, and Mechanistic Analysis of Intellectual Disability Syndrome Protein ABBA
Source: Mol Neurobiol. 2025 Dec 8;63(1):271. doi: 10.1007/s12035-025-05475-3 (PMC12686055; doi:10.1007/s12035-025-05475-3)

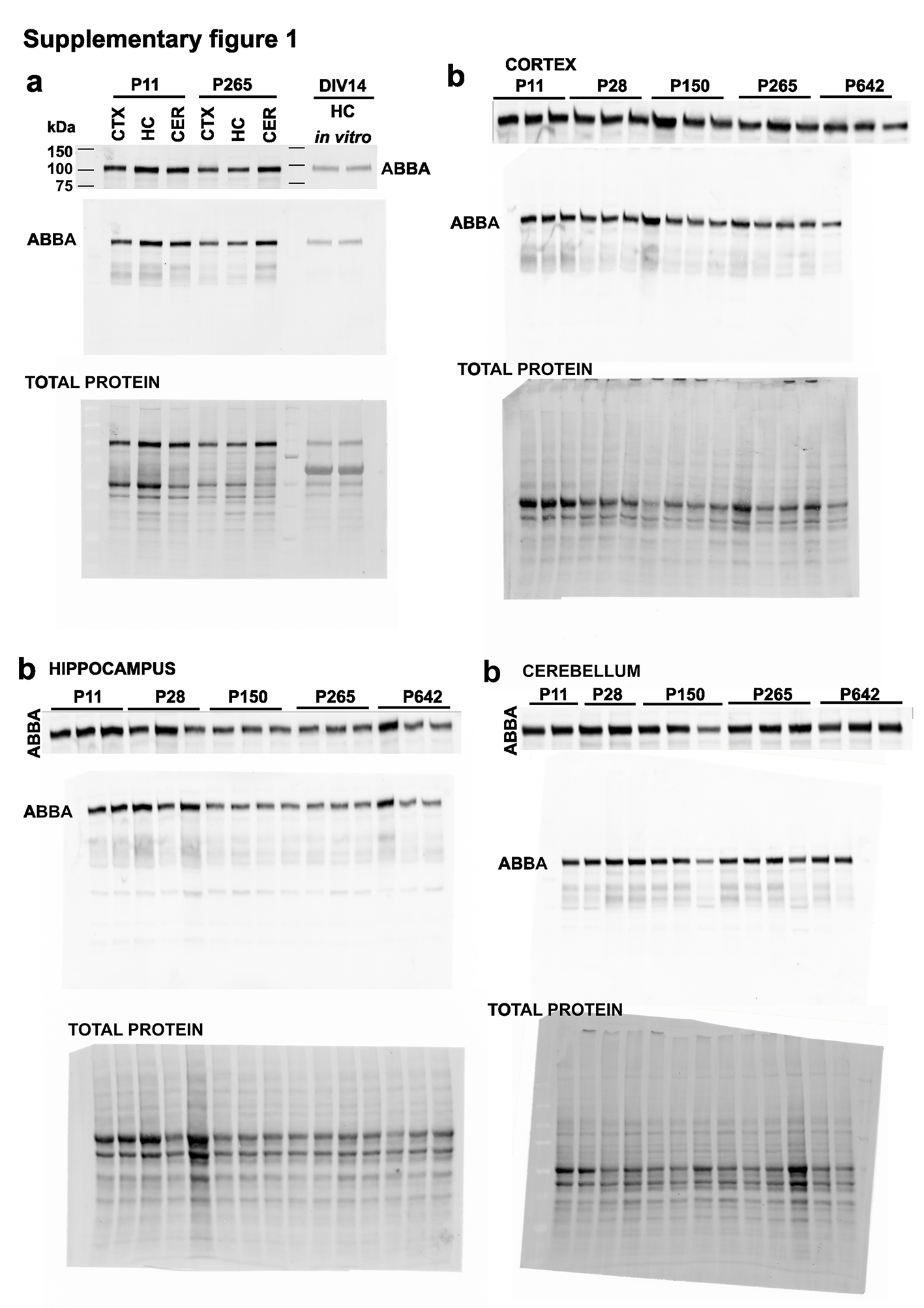

Supplement: Supplementary file 1 — (PNG 863 KB) [file 12035_2025_5475_Fig9_ESM.png]

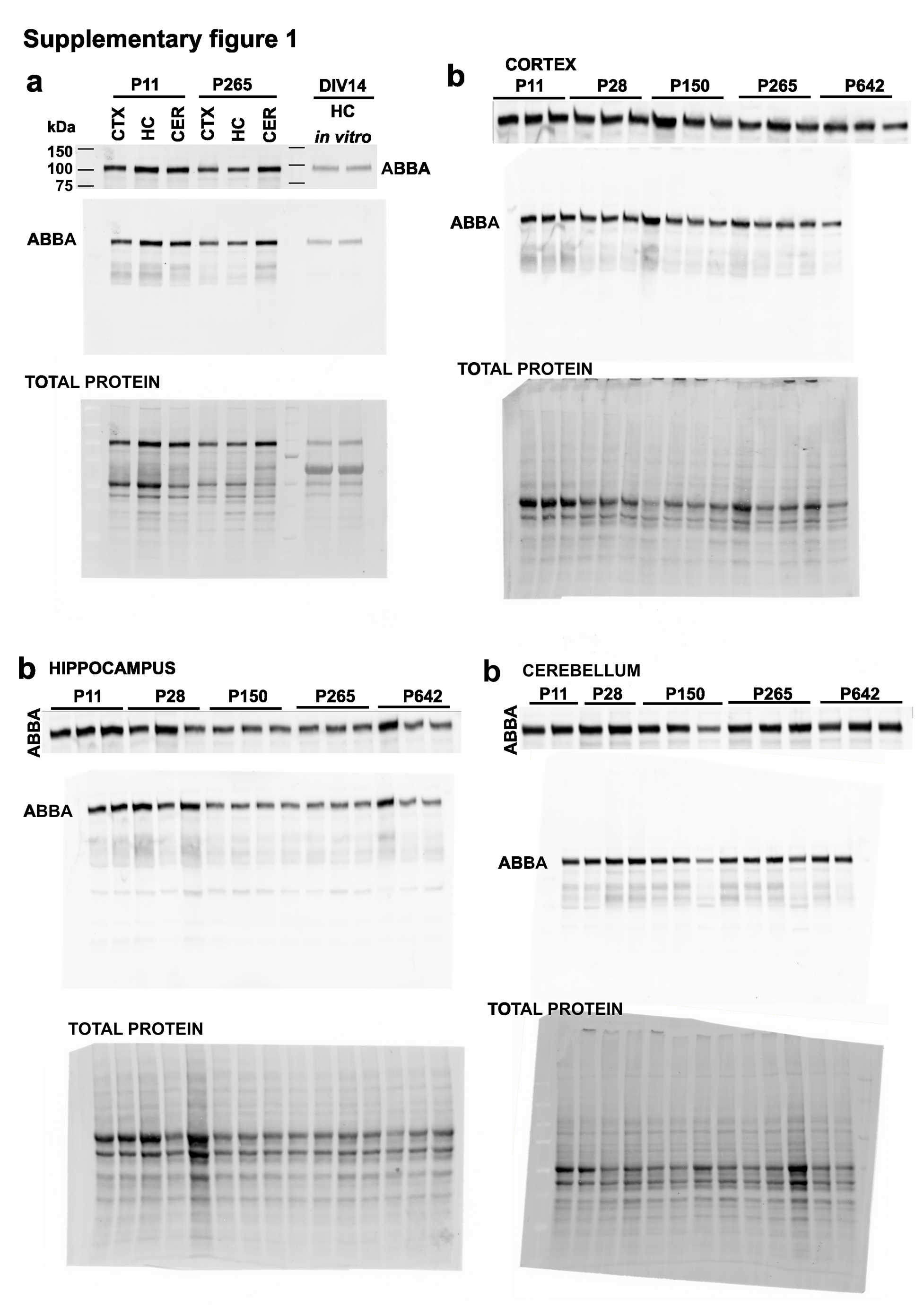

Supplement: Supplementary file 2 — (TIF 15.4 MB) [file 12035_2025_5475_MOESM1_ESM.tif]

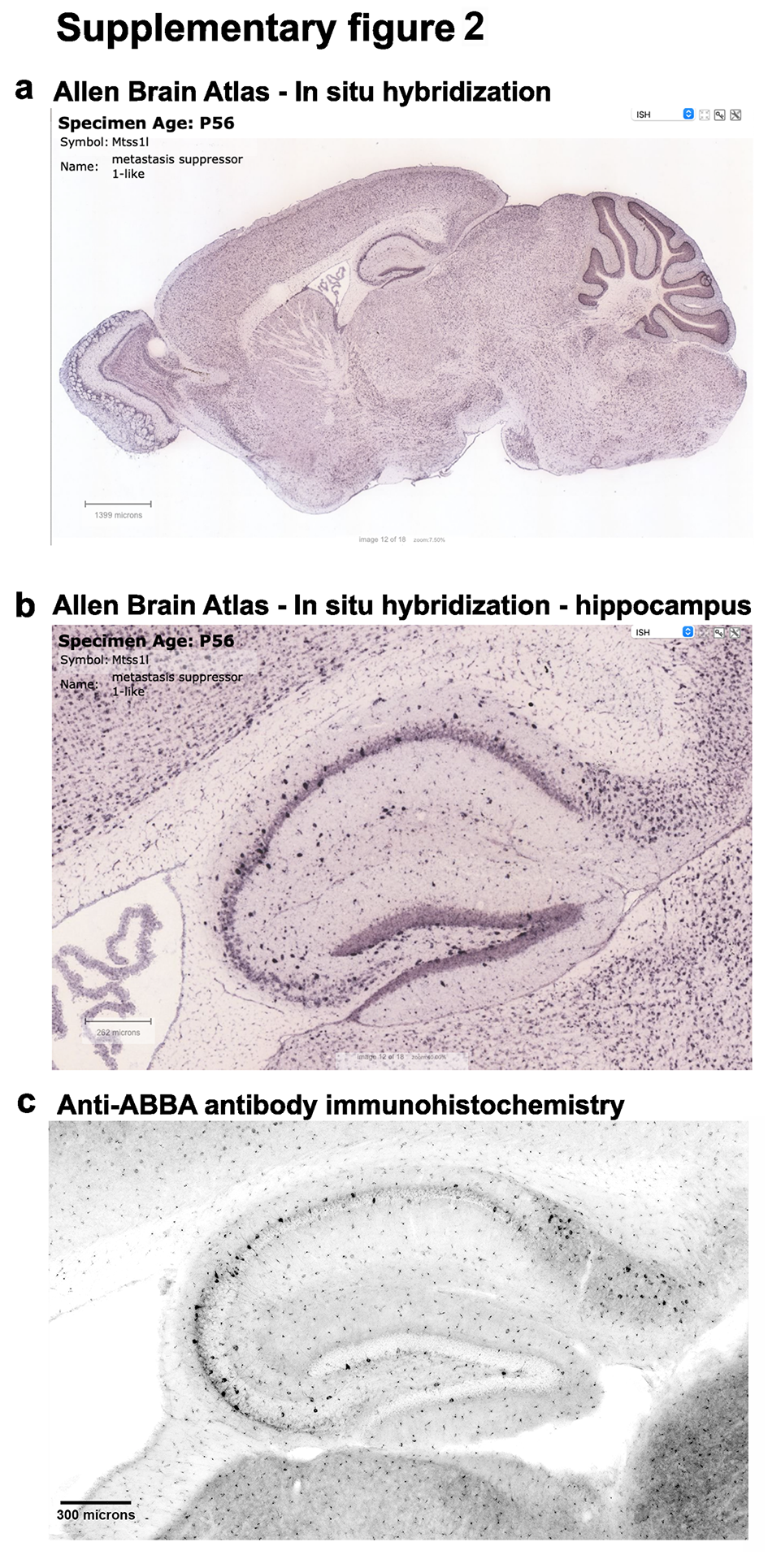

Supplement: Supplementary file 3 — (PNG 1.82 MB) [file 12035_2025_5475_Fig10_ESM.png]

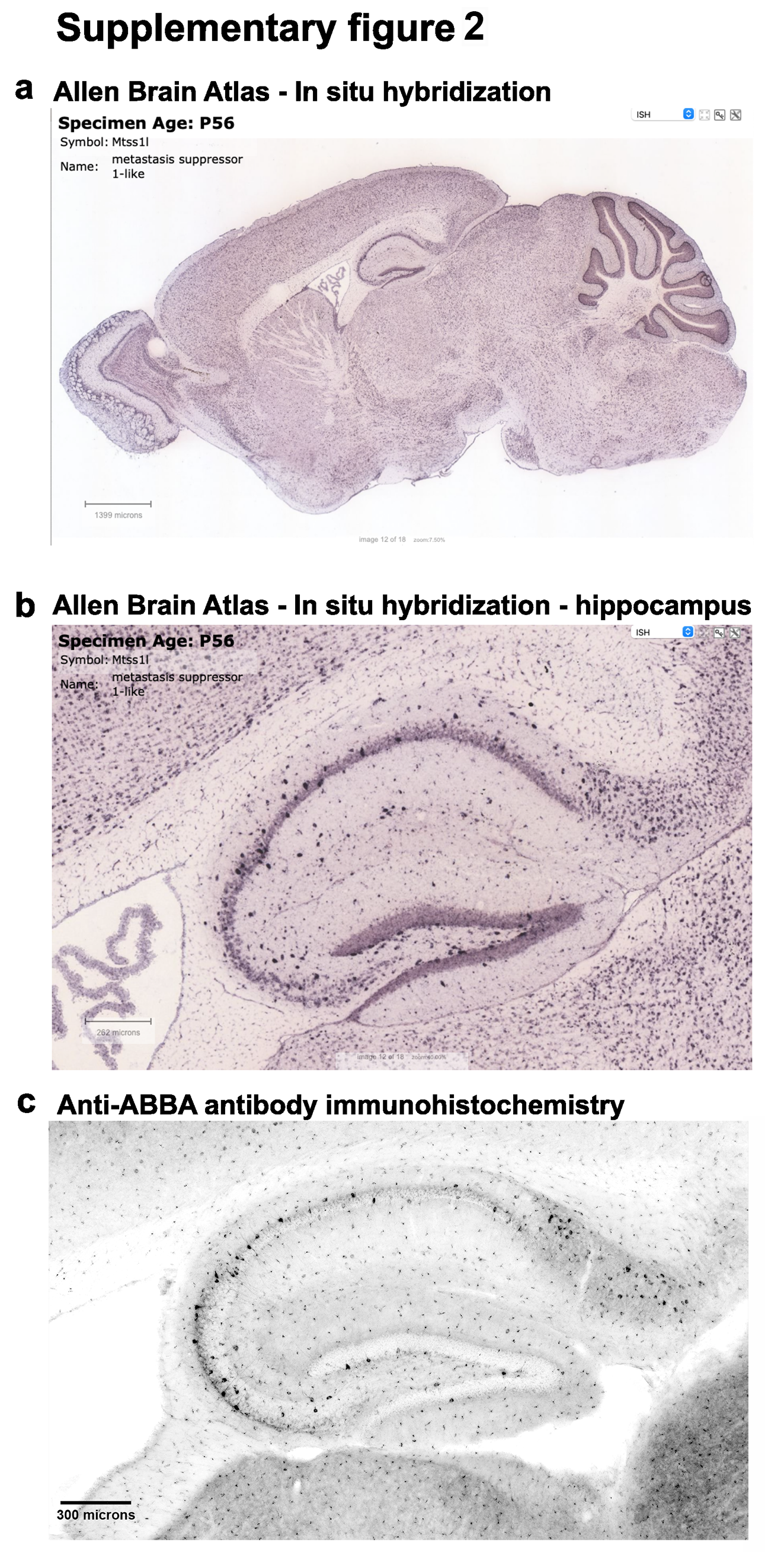

Supplement: Supplementary file 4 — (TIF 18.7 MB) [file 12035_2025_5475_MOESM2_ESM.tif]

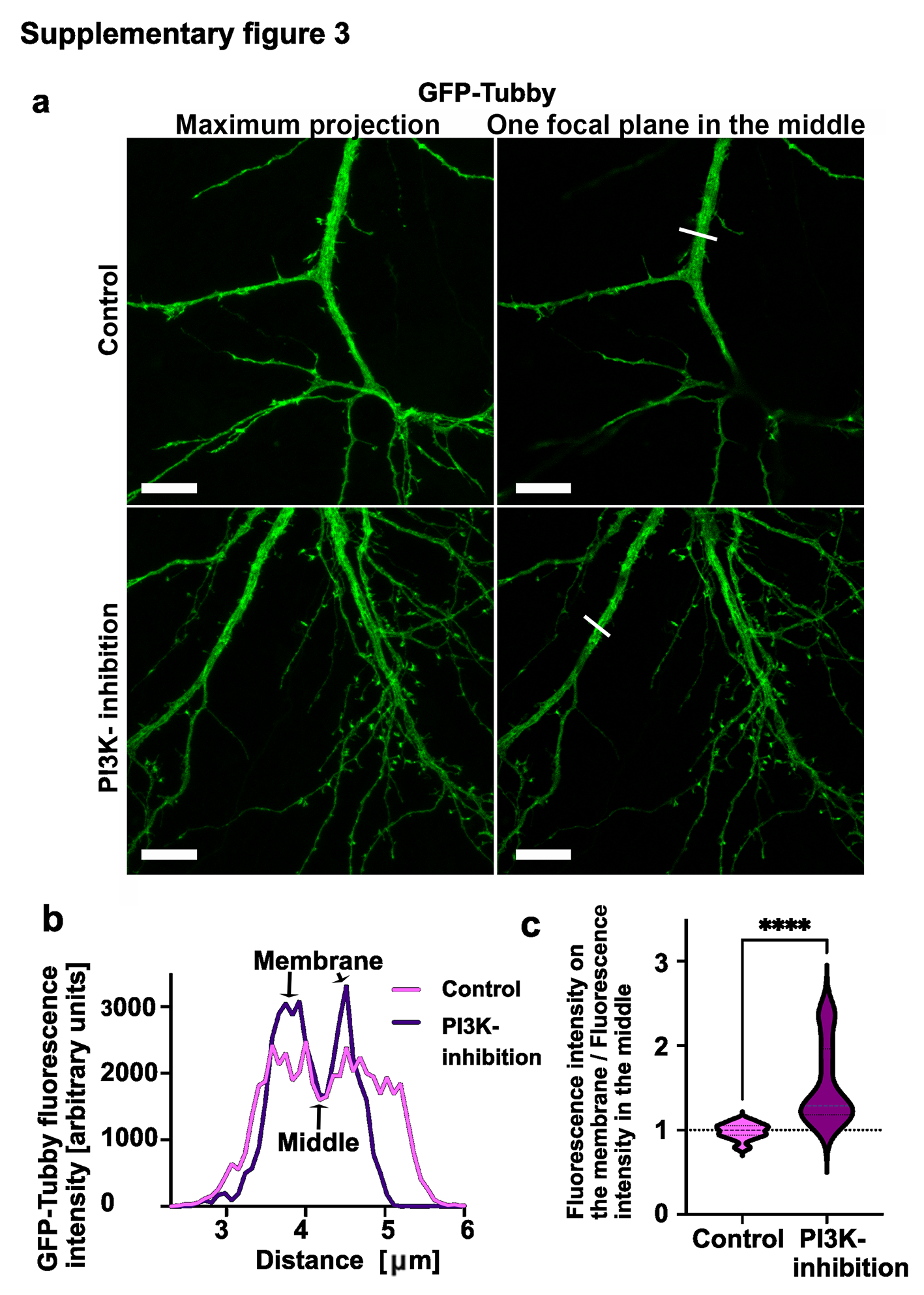

Supplement: Supplementary file 5 — (PNG 1.04 MB) [file 12035_2025_5475_Fig11_ESM.png]

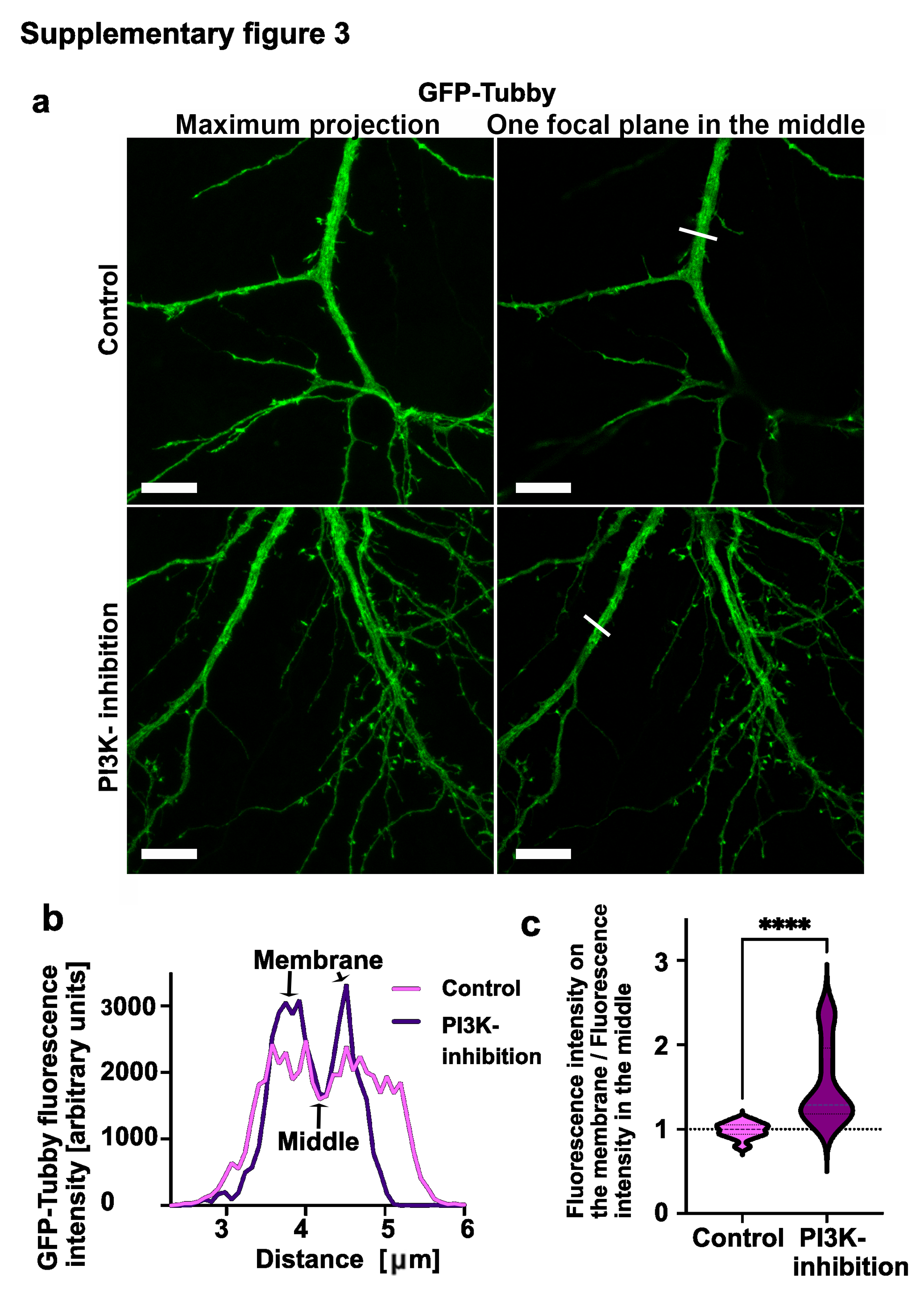

Supplement: Supplementary file 6 — (TIF 15.6 MB) [file 12035_2025_5475_MOESM3_ESM.tif]

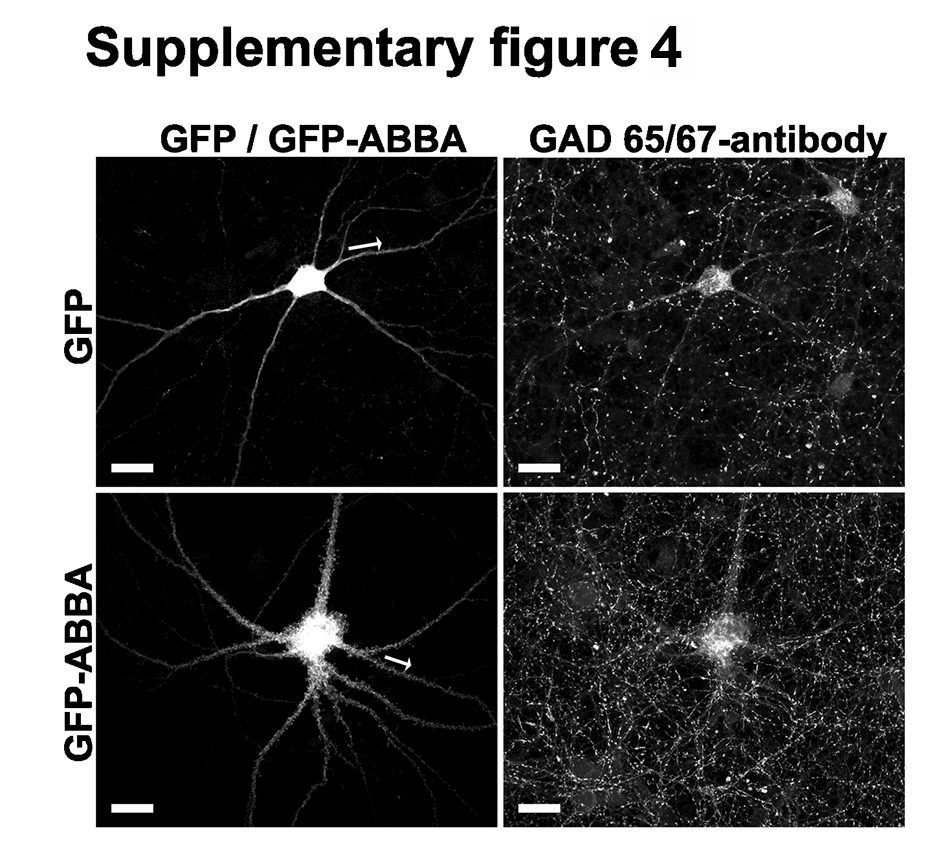

Supplement: Supplementary file 7 — (PNG 443 KB) [file 12035_2025_5475_Fig12_ESM.png]

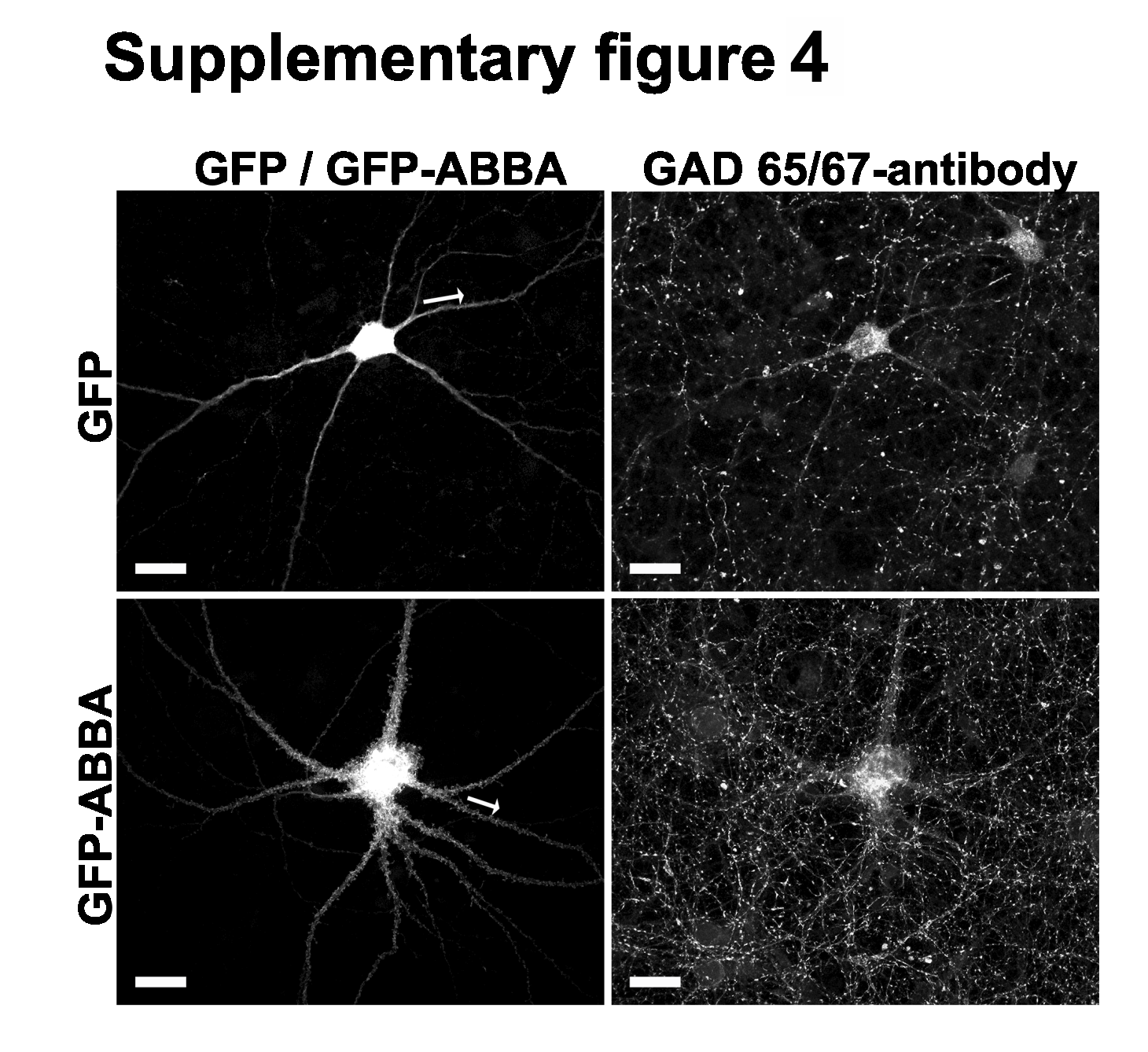

Supplement: Supplementary file 8 — (TIF 8.04 MB) [file 12035_2025_5475_MOESM4_ESM.tif]
