# Supplementary material for: Expression, Subcellular Localization, and Mechanistic Analysis of Intellectual Disability Syndrome Protein ABBA
Source: Mol Neurobiol. 2025 Dec 8;63(1):271. doi: 10.1007/s12035-025-05475-3 (PMC12686055; doi:10.1007/s12035-025-05475-3)

Supplementary information: Uncropped Western blots presented in Fig 1.

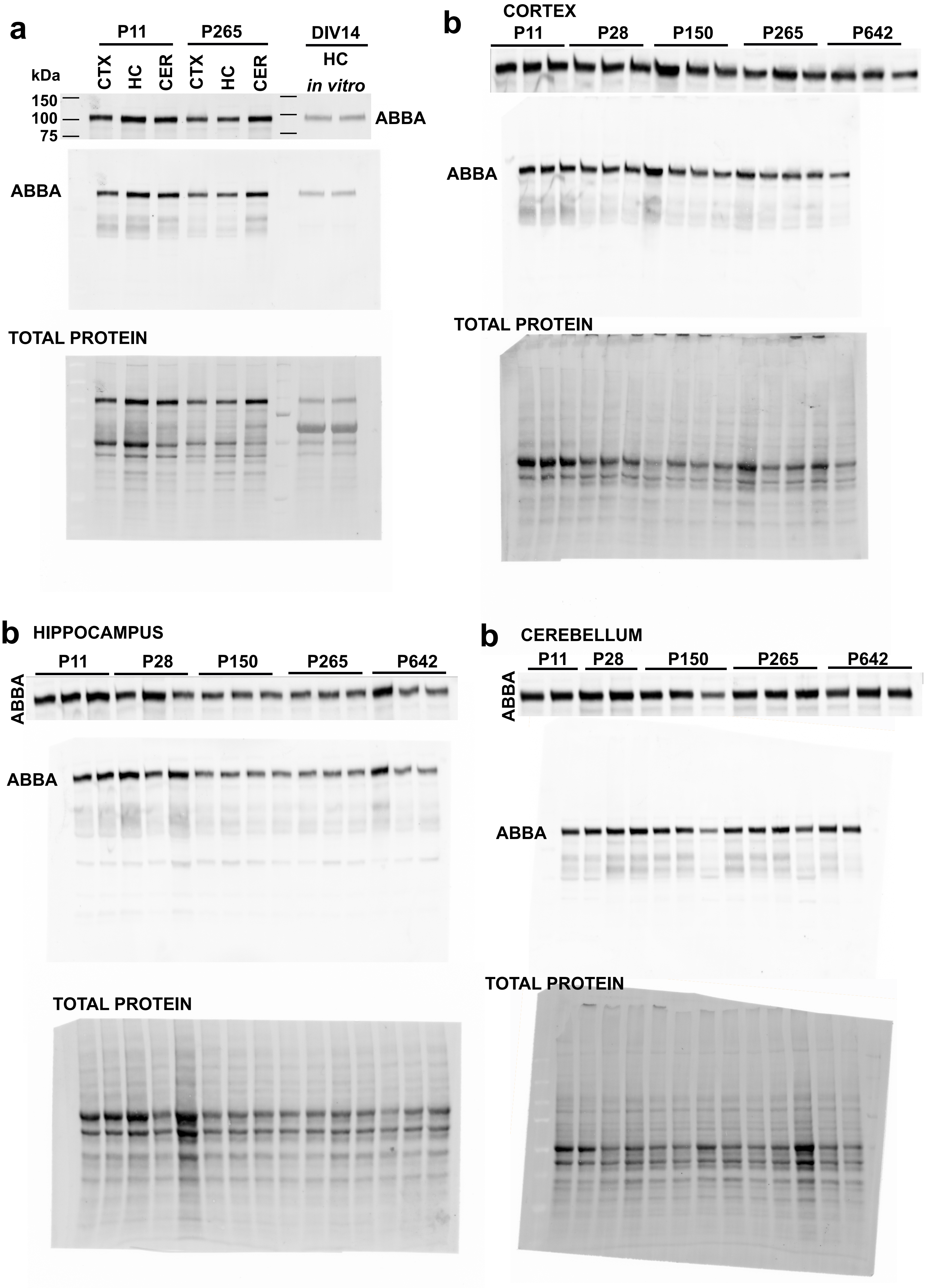

Supplement: Supplementary file 9 — (PDF 2.71 MB) [file 12035_2025_5475_MOESM5_ESM.pdf]
